# Applying therapist-guided digital cognitive behavioral therapy for insomnia in psychiatry: a mixed-methods process evaluation

**DOI:** 10.1186/s12888-025-06824-1

**Published:** 2025-04-28

**Authors:** J. E. Reesen, F. M. van de Kamer, A. E. van Keeken, S. L.C. Ikelaar, P. van Oppen, N. Batelaan, J. Lancee, E. J.W. van Someren, F. van Nassau

**Affiliations:** 1 Department of Sleep and Cognition, Netherlands Institute for Neuroscience, Royal Netherlands, Academy of Arts and Sciences, Amsterdam, the Netherlands; 2 Department of Integrative Neurophysiology, Center for Neurogenomics and Cognitive Research, Amsterdam Neuroscience, Vrije Universiteit University Amsterdam, Amsterdam, the Netherlands; 3 Department of Psychiatry, Amsterdam UMC location Vrije Universiteit Amsterdam, Amsterdam, the Netherlands; 4 GGZ inGeest Mental Health Care, Amsterdam, the Netherlands; 5 Department of Psychiatry, Amsterdam Public Health research institute, Amsterdam University Medical Center, Vrije Universiteit, Amsterdam, The Netherlands; 6 Department of Clinical Psychology, University of Amsterdam, Amsterdam, The Netherlands; 7 Amsterdam Neuroscience, Mood Anxiety Psychosis Stress Sleep, Amsterdam, the Netherlands; 8 Department of Public and Occupational Health, Amsterdam Public Health research institute, Amsterdam University Medical Center, Vrije Universiteit, Amsterdam, the Netherlands

**Keywords:** Process evaluation, Insomnia, CBT-I, Hybrid type 2

## Abstract

**Introduction:**

Insomnia is prevalent, particularly among individuals with mental health complaints. However, Cognitive Behavioral Therapy for Insomnia (CBT-I), the first-line treatment, is underutilized in care settings. This study evaluated a therapist-guided digital CBT-I (i-Sleep), gathering insights from participants and therapists to optimize the intervention and inform implementation strategies.

**Methods:**

A mixed-methods process evaluation, guided by the RE-AIM framework, was conducted alongside an effectiveness trial. Data were collected from i-Sleep participants with clinically relevant insomnia and various mental health complaints across all care levels, ranging from pre-clinical (unattended), to those referred to general or specialized care. Additionally, data were collected from i-Sleep therapists.

**Results:**

A total of 181 i-Sleep participants (mean age = 46.7 years, SD = 13.2) enrolled, with an attrition rate of 21.6%. Participants reported benefits including faster sleep onset, fewer nighttime awakenings, increased daytime energy, and positive lifestyle changes, though some experienced minimal gains or adverse effects. Satisfaction with the intervention ranged from 7.1 to 7.3 across care levels. Post-intervention, 89.4% of all participants indicated they would recommend iCBT-I. Satisfaction with therapist guidance was high (M = 7.7–8.3), though preferences for format and frequency varied. Therapists (*n* = 15, mean experience = 0.8 years, SD = 1.1) suggested addressing practical constraints and enhancing training for better integration into routine care.

**Conclusion:**

Our findings highlight the feasibility and potential of therapist-guided iCBT-I to improve sleep in individuals with mental health complaints across all care settings. Universal implementation could offer significant benefits, while adaptable content and flexible guidance may better meet individual needs.

**Trial registration:**

Netherlands Trial Register (NL9776) registered on 07/10/2021.

**Supplementary Information:**

The online version contains supplementary material available at 10.1186/s12888-025-06824-1.

## Background

Insomnia is defined by persistent difficulties in initiating or maintaining sleep, or waking up too early, accompanied by significant subjective daytime impairments such as fatigue, cognitive difficulties, and mood disturbances, occurring despite adequate opportunity for sleep [1]. Affecting approximately 10% of adults globally, insomnia imposes a substantial burden on individuals [2]. Insomnia is often chronic, with a 40% persistence rate over a 5-year period. It affects certain populations more severely, including women, older adults, and those facing socioeconomic hardships [2]. Insomnia not only undermines quality of life and daily functioning but also significantly increases the risk of various health issues, including cardiovascular disease, diabetes, and especially mental health disorders [3–5]. Notably, insomnia frequently co-occurs with mental health conditions such as depression, anxiety disorders, and post-traumatic stress disorder (PTSD), with almost 70% of individuals with mental disorders reporting sleep difficulties and over 30% meeting the criteria for insomnia disorder [6]. This overlap worsens these conditions and complicates treatment outcomes, as patients with comorbid insomnia often experience a more adverse course of their mental disorder and reduced treatment efficacy [7, 8]. Addressing insomnia is crucial not only for improving sleep but also for mitigating its broader impact on mental health and overall well-being.

Cognitive Behavioral Therapy for Insomnia (CBT-I) is the first-line treatment for insomnia [1, 9]. Research has consistently demonstrated its moderate to large effects, leading to clinically significant improvements in insomnia severity [10, 11] that endure for up to a year post-therapy [12]. Beyond its primary role in treating insomnia, CBT-I has also shown promise in alleviating comorbid mental health disorders such as depression and PTSD [13] and recent studies further emphasize its role in anxiety improvement [14–16]. This underscores the critical need for accessible CBT-I in mental healthcare settings.

Despite its proven efficacy, the integration of CBT-I into routine clinical practice remains limited, leaving significant gaps in accessibility for those who need it. Several barriers hinder its widespread adoption: (a) systemic challenges such as insurance deductibles and referral requirements; (b) clinician-related issues like inadequate training and limited familiarity with non-pharmacological treatments for insomnia, and (c) patient-related obstacles, including lack of awareness and difficulties in accessing care [17–19].

A potential solution to overcome (some) of these barriers may lie in digital formats of CBT-I (iCBT-I), which can be fully automated or therapist-guided. While both formats improve scalability, accessibility, and convenience, guided iCBT-I may offer middle ground by balancing the efficiency of digital automation with the adaptability of therapist oversight. Evidence suggests that guided iCBT-I is non-inferior to traditional face-to-face CBT-I, effectively reducing insomnia severity with sustained improvements over time [20]. Several studies indicate that guided iCBT-I it can be delivered effectively by providers with less specialized training, such as GP nurses [21], thus reducing the need for highly trained specialists. This flexibility decreases the direct clinician time required, lowers training demands, and helps alleviate workforce shortages. Additionally, its structured digital format may streamline insurance reimbursement and referral pathways. At the same time, guided iCBT-I overcomes key drawbacks of fully automated programs by incorporating therapist oversight, thereby allowing for tailored feedback, greater adaptability to individual patient needs, and improved engagement [22]. Despite these advantages, also guided iCBT-I has faced slow uptake in routine care, highlighting the need for a deeper investigation into the facilitators and barriers to its implementation, the mechanisms, and experiences surrounding the intervention.

One example of such an iCBT-I intervention is i-Sleep. Previous research has demonstrated the efficacy of i-Sleep across diverse populations [21, 23, 24]. Currently, we are conducting the *‘Better nights, better days?’* study to assess the effectiveness of i-Sleep in individuals who are experiencing clinically significant insomnia, along with symptoms of generalized anxiety disorder (GAD), social anxiety disorder (SAD), panic disorder (PD), PTSD, obsessive-compulsive disorder (OCD) or borderline personality disorder (BPD) [25, 26]. This randomized controlled trial (RCT) encompasses individuals across all levels of mental health care, from those unattended with early symptoms (pre-clinical) to those awaiting treatment in general or specialized mental health care settings. This design provides a unique opportunity to explore variations in experiences, views, suggestions, and barriers across different levels of mental health care.

In this study on i-Sleep, we conducted an extensive process evaluation, as part of a hybrid type II effectiveness trial [27], with efficacy outcomes reported separately. Our evaluation was guided by the RE-AIM framework, which covers five key dimensions: (1) *Reach*, examining reasons for participation or dropout, and participant demographics; (2) *Effectiveness*, assessing the intervention’s impact on sleep quality, participant satisfaction, and overall well-being; (3) *Adoption*, exploring therapists’ characteristics and their motivations for either adopting or discontinuing the intervention; (4) *Implementation*, evaluating participant adherence, therapist protocol fidelity, and the use of iCBT-I training and support resources among therapists; and (5) *Maintenance*, focusing on the long-term use of the intervention by participants and therapists. Building on this framework, we aim to pinpoint specific implementation challenges and opportunities. Ultimately, our goal is to optimize the therapist guided iCBT-I intervention and inform implementation processes to enhance its integration in real-world mental health care settings.

## Methods

This process evaluation utilizes a mixed-methods design guided by the RE-AIM framework [28]. Table 1 summarizes the objectives, methods, and operationalization of the framework at each time point. Results will also be structured around the dimensions of the RE-AIM framework. The COREQ checklist is presented in the appendix.

**Table 1:** Overview of process evaluation objectives and methods using the RE-AIM framework *Abbreviations. Q = Questionnaire*

	Timepoint		Participants											Therapists			
			Baseline		During intervention			Follow-up assessments					Enrollment			Continuous	
			T_0_		T_intervention_			T1			T2		-t_1_			T_cont._	
	*Week number*		*1*		*3–7*			*11*			*37*						
	*Data source / method*		*Q*		*Exit Interview*	*Sleep Registry*		*Interview*	*Q*		*Q*		*Q*			*Focus group*	*Log-book*
*Reach*	Reasons for joining, continuing with, or opting out of the intervention among participants		X		X			X									
	Participant characteristics, e.g., demographics and mental health		X						X		X						
*Effectiveness*	Perceived effectiveness of the intervention on sleep quality, sleep environment and quality of life							X	X		X						
	Satisfaction, views, and experiences with the intervention among participants				X			X	X		X					X	
*Adoption*	Therapists’ characteristics, e.g., demographics, skills, and experience												X				
	Reasons among supervising therapists/ experts for joining, continuing with, or opting out of using the intervention												X			X	
*Implementation*	Treatment adherence among participants, e.g., completed sessions and assignments (fidelity)					X		X	X		X						
	Degree in which the intervention protocol has been followed by therapists (fidelity and dosage)																X
	Use (dosage and fidelity) of provided training/support regarding the intervention among therapists															X	X
	Satisfaction, views and experiences with the intervention and provided training/support among therapists												X			X	
*Maintenance*	Long-term helpfulness and continued usage of intervention elements among participants																
	Intention for long-term use of the intervention among therapists							X			X						
	Future activities and intention to use the intervention of therapists/ patients?							X			X						
*Overall*	Recommendations for improvement of the intervention and implementation support beyond the research setting							X								X	X
	Perceived barriers and facilitators to the adoption, implementation and continuations of the intervention							X								X	X

### The *‘Better nights, better days?’* study

This study employs a Hybrid Type 2 design, with process evaluation and efficacy results reported separately (see Appendix for a flowchart outlining the allocation of efficacy and process evaluation measures and results). A detailed description of the study has been provided elsewhere [25, 26]. In short, the *‘Better nights, better days?’* study is a transdiagnostic and pragmatic RCT with randomization at the patient level and implemented across all echelons of mental health care. Participants were recruited via multiple strategies. Individuals from the preclinical group were primarily recruited through newsletter invitations from the Netherlands Sleep Registry. Participants awaiting mental healthcare were recruited pragmatically, making effective use of their waiting-list time, through outpatient clinics and general practitioner (GP) practices (e.g., GGZ InGeest). Additional methods included flyers distributed in clinics and practices, as well as broader outreach via media channels and medical professional networks. The targeted mental health care (MHC) levels include: 1) people with pre-clinical complaints from the general population not currently in or awaiting mental health treatment (pre-clinical), 2) people referred by their GP to general MHC, awaiting diagnosis and treatment (or in early stages of treatment), and 3) people with a GAD-, SAD-, PD-, PTSD- or OCD diagnosis referred to specialized MHC, awaiting regular treatment (or in early stages of treatment). After baseline assessments, recruited participants were randomized to either iCBT-I or waitlist-control. Extensive sleep and mental health assessments took place at baseline (T0), post-test after completion of the intervention (T1; T0 + 2 m), and at follow-up, six months later (T2; T0 + 8 m). The study was approved by the Medical Ethics Review Committee of the VU University Medical Center Amsterdam Centre (METC VUmc, registration number 2021/093), and is registered as a clinical trial on the Netherlands Trial Register (NL9776), with the registration date of 07/10/2021. All participants provided informed consent and received €80 in financial compensation upon completion of the study.

### Participants

This process evaluation included participants from the *‘Better nights, better days?’* study who were randomized to the i-Sleep condition, as well as the therapists delivering the i-Sleep intervention. The inclusion criteria for the participants were: (1) adults aged 18 years or older; (2) clinically relevant insomnia symptoms, defined by an Insomnia Severity Index (ISI) score of 10 or greater [29]; (3) clinically relevant complaints as assessed by the Rapid Measurement Toolkit 20 (RMT-20 [30]) or the Ultra-Short Borderline Personality Disorder Checklist (BPD-C [31, 32]), meeting at least one of the following subscale cut-offs: PTSD (≥ 8), SAD (≥ 12), PD (≥ 9), GAD (≥ 11), and/or BPD (≥ 14); (4) able to complete online questionnaires and diaries in Dutch. Solely people recruited through specialized MHC required meeting the criteria for GAD-, SAD-, PD, PTSD or OCD according to the Mini International Neuropsychiatric Interview (M.I.N.I.) [33]. The exclusion criteria were: (a) alcohol or substance dependency, as assessed by the Alcohol Use Disorders Identification Test (AUDIT) (≥ 20 [34]) and Drugs Use Disorders Identification Test (DUDIT) (≥ 25 [35]); (b) a current diagnosis of bipolar or psychotic disorder; (c) prior receipt of (i)CBT-I treatment, either currently or within the past three months. Sleep apnea, use of sleep medication, or shift work were not exclusion criteria, as these factors do not necessarily interfere with the benefits of CBT-I [36–38]. See Reesen et al. [26] for more details. i-Sleep therapist recruitment was pragmatic, focusing on researchers from the *‘Better nights*,*better days?’* study and trained CBT-I psychologists from prior collaborations. Therapists had to complete an i-Sleep training session before delivering guidance.

### The therapist guided iCBT-I intervention (i-Sleep)

The therapist guided iCBT-I intervention, known as i-Sleep, is delivered in five sequential sessions over 5 to 8 weeks [39]. In this study, we utilized an adapted i-Sleep version for mental health care, which included case vignettes depicting patients with comorbid psychiatric symptoms. See Table 2 for a more detailed description of the content. Each week, participants complete one session containing informative texts, experiential videos, guiding questions, and (audio)exercises. Over the remainder of the week, they engaged daily with the session material, typically for 10 to 30 min per day, by maintaining a daily sleep diary and practicing the exercises (e.g.: relaxation techniques or restructuring negative beliefs). Upon finishing the final session, participants received a downloadable version of i-Sleep for continued use. Therapists provided personalized online written feedback after each session to motivate participants and guide them through the intervention. They offered practical advice, and if needed slightly tailored strategies to each participant’s specific needs (e.g., determining appropriate sleep windows, challenging persistent negative beliefs). Sleep compression was not used as a formal alternative to sleep restriction. Therapists were trained in a 2-hour training session led by experienced i-Sleep practitioners (JL, JR) and received intensive supervision for at least the first participant. After demonstrating proficiency, they proceeded independently but were required to attend weekly collaborative case discussions to discuss treatment progress. Therapists had access to feedback templates, and experienced i-Sleep practitioners were available for consultation. The intervention was facilitated on the Netherlands Sleep Registry (www.slaapregister.nl) and was provided in a research setting.

**Table 2:** Content overview of sessions guided iCBTI intervention i-Sleep

Session		Theme		Procedures
1		Psychoeducation on sleep, disordered sleep and sleep hygiene		General guidelines about health and environmental factors influencing sleep are provided.
2		Sleep restriction and stimulus control training		For the sleep restriction: initial sleep window generation is the average total sleep time (TST) based on a one-week sleep diary (self-reported) with a minimal sleep window of five hours. The position of the sleep window is based on patient preference and tailored to their daily schedule. After a week, the sleep window is extended with 15 min when sleep efficiency (SE) ≥ 85%. The prescribed time in bed remains unadjusted if SE < 85%. Participants are also strongly advised against daytime napping, yet if this is not feasible for the participant daytime napping is limited to 20–30 min. The association of bed with sleeping is reinforced during the stimulus control training. Participants are instructed to reserve their bed for sleep and sex alone and are motivated to leave their bed after lying awake for 15 to 30 min.
3		Rumination and relaxation techniques		Various techniques are introduced: a 15-minute worry period; focusing on staying awake; blocking thoughts by continuously thinking of something else; and (audio) relaxation exercises.
4		Cognitive restructuring		To change misconceptions about sleep, participants are instructed to fill out thought-schemes and to change non-helping thoughts.
5		Relapse prevention		Summary of all prior sessions is provided. Participants are invited to reflect on their progress and instructed to create a toolbox/plan to prevent future relapse.

### Process evaluation data collection

Data collection for the process evaluation occurred between December 2021 and August 2024. Data was gathered from both i-Sleep participants and therapists using a combination of questionnaires interviews and focus groups, as summarized in Table 1. All questionnaires were completed online via the platform Netherlands Sleep Registry. Interviews and focus groups followed semi-structured guides based on the RE-AIM framework (see Appendix). An overview of all administered process evaluation questionnaires, including specific items and scales, is available in the Appendix.

#### i-Sleep participants

For this process evaluation, a total of 181 participants were randomized to the iCBT-I intervention arm. Process evaluation questionnaires were administered at two key time points: post-intervention (T1) and follow-up (T2). These questionnaires specifically assessed participant experiences with the iCBT-I intervention elements, focusing on three key aspects measured through 10-point Likert scales: (a) *adherence*, i.e. the extent to which participants followed the recommended guidelines and completed the prescribed activities of the iCBT-I intervention (1 = never performed, 10 = performed daily); (b) *feasibility*: i.e. how manageable and achievable the participants considered it to integrate the advice and tasks of the iCBT-I intervention into their daily lives (1 = not at all feasible, 10 = highly feasible, NA = not applicable); and (c) *perceived effectiveness*: i.e. the degree to which participants felt that the advice and techniques of the iCBT-I intervention successfully reduced their sleep complaints (1 = not at all effective, 10 = highly effective, NA = not applicable). Participants also evaluated the therapist guidance, measuring the sufficiency of personalized guidance (yes, sufficiently personalized / no, insufficiently personalized) and assessing the quality of advice, consideration of personal circumstances, therapist availability, provision of options, progress guidance, motivation, instilling confidence, and whether the therapist took enough time using a 10-point Likert scale (1 = completely disagree, 10 = completely agree).

Next to the questionnaires, telephone interviews were conducted with a purposively selected subset of participants (*n* = 36). Selection criteria included varying session completion (1–5), demographics (age, sex, education level), and baseline symptoms of insomnia (ISI score) and mental health (RMT20, BPD-C scores). The aim was to capture a diverse range of experiences to ensure a comprehensive understanding of potential variations in participant perspectives. This approach maximized variation to provide a nuanced understanding of participant experiences. Participants were approached via telephone and mail. The interviews, conducted by FVK and AVK between August 2022 and July 2024, aimed to capture detailed insights into participant experiences with the intervention. Exit interviews were conducted with participants who chose to discontinue i-Sleep to collect their reasons for dropping-out.

The data collected for process evaluation were complemented with baseline data from the RCT effectiveness study [26], which included demographic information age, sex, and clinical diagnosis assessed using the M.I.N.I and baseline symptom severity for insomnia and mental health, evaluated through validated questionnaires such as the ISI, RMT20, and BPD-C.

#### i-Sleep therapists

All fifteen therapists guiding participants in the i-Sleep program completed a questionnaire on their initial perspectives and motivations using 5-point Likert scales (1 = strongly disagree, 5 = strongly agree), their training experiences using 10-point Likert scales (1 = completely unprepared, 10 = very well prepared) and open-ended responses, as well as demographic information (see Appendix). Additionally, therapists maintained detailed logbooks documenting their interactions with participants, including time spent on guidance for each session and the number of reminders sent. This guidance encompassed activities such as reviewing session responses, monitoring bedtimes and sleep efficiency scores, providing feedback, and sending reminders for incomplete sessions. Online focus group discussions, held in February–March 2023 and facilitated by FVN and AVK, included all nine therapists actively engaged in the intervention at that time. Therapists who joined the program later were not part of these discussions.

The researchers who conducted the interviews and focus groups were all female, holding a PhD, MSc, or BSc, and were well-trained in qualitative research. AVK had a coordinating role during the measurements of the participants.

### Data analysis

#### Quantitative data

Descriptive statistics (mean, SD) were used to summarize participants’ and therapists’ characteristics as well as self-reported questionnaire data and logbooks of therapists. Descriptive statistics were conducted using R statistical software (version 4.2.2 [40]).

#### Qualitative data

All interviews and focus groups were audiotaped, fully transcribed verbatim and anonymized. Participants were not provided with the transcripts for review or correction. A codebook was developed and refined through a codebook session (FVK, FVN, JR), which provided a structured framework for analysis. Coding was conducted in MAXQDA 2024 [41]. Transcripts were analyzed using a direct content analysis approach, applying the finalized codebook to address each pre-specified research question (Appendix). Texts that could not be categorized within the initial coding scheme were assigned new open codes inductively. The coded data were then thematically analyzed and integrated into the RE-AIM model [28]. Appropriate quotes were selected to illustrate key themes and provide deeper insights into the data.

#### Triangulation and data synthesis

Qualitative and quantitative data were gathered simultaneously. Two researchers (JR, FVK) independently coded the data throughout the study. This coding was then compared to determine if insights from qualitative sources could complement or contradict the observations from quantitative data, to validate findings across various data source.

## Results

This section follows the RE-AIM dimensions; Reach, Effectiveness, Adoption, Implementation, and Maintenance. Participant data were analyzed across the three levels of mental health care: pre-clinical, general, and specialized. The results conclude with recommendations for optimizing the i-Sleep intervention and facilitating its implementation beyond the current research setting.

A total of 181 participants of the ‘*Better nights*,* better days?*’ study were randomized into the i-Sleep condition, all of whom completed the baseline questionnaires. This group consisted of 80 participants in the preclinical group, 42 in general-, and 59 in specialized MHC group. Notably, data collection is ongoing for the latter two groups; specifically, T2 assessments are still pending for 10 participants in the general MHC group and 18 participants in the specialized MHC group. In addition to the questionnaires, a total of 36 interviews were conducted with a subset of i-Sleep participants, averaging 38 min in duration (range: 19–61 min). Fifteen therapists provided guidance in the study, all of whom completed a survey regarding the intervention. Among these therapists, nine participated in focus group sessions, which comprised a total of three sessions with an average duration of 53 min (range: 49–57 min).

### RE-AIM: the reach dimension

The Reach dimension concerns reasons for joining, continuing, or opting out of the iCBT-I intervention, as well as participant characteristics, including demographics and mental health complaints.

#### Participant demographics and reasons for engagement or discontinuation in the CBT-I intervention

The average age of participants across all levels of mental health care was 46.7 years (SD = 13.2), and 75.1% was female. Table 3 provides an overview of participant characteristics. Most participants entered the study hopeful for improvement in their insomnia but often lacked specific expectations. They sought practical advice and personalized strategies. One participant noted, *“I didn’t have any specific expectations right away. I did hope to gain some tools to help with being less tired during the day by improving my sleep. Beyond that*,* I didn’t have any particular expectation”*– Participant 1 (pre-clinical group). Others approached the intervention skeptically, with one stating, *A bit blank in the sense that I had good hope this could bring something*,* but also because of past experiences*,* I didn’t let my hope get too high because I know it’s not an easy task when you’ve been sleeping poorly for so long”*– Participant 2 (pre-clinical group).

**Table 3:** Baseline (T0) characteristics of i-Sleep participants across the pre-clinical, general- and specialized mental health care groups

				Mean (SD) or *n* (%)							
				Pre-clinical group			General MHC group			Specialized MHC group	
				All (*n* = 80)	Interviewed participants (*n* = 11)		All (*n* = 42)	Interviewed participants (*n* = 13)		All (*n* = 59)	Interviewed participant (*n* = 12)
*Demographics*	Female			57, 71.3%	9, 81.8%		32, 76.2%	10, 76.9%		46, 78.0%	9, 75.0%
	Age (years)			54.1 (13.8)	55.1 (14.7)		43.2 (13.2)	41.4 (10.9)		42.7 (12.4)	40.4 (11.2)
	Medication use^*^ *ever* *past two months*			51 (63.8%) 19 (23.8%)	8 (72.7) 3 (27.3)		23 (54.8%) 14 (33.3%)	7 (53.8%) 5 (38.5%)		47 (79.7%) 31 (52.4%)	10 (83.3%) 5 (41.7%)
*Sleep*	Insomnia severity (ISI)			17.4 (4.1)	18.5 (4.2)		16.6 (3.7)	17.0 (4.5)		17.9 (5.0)	20.1 (3.9)
	Narcolepsy			8 (10.0%)	1 (9.1%)		3 (7.1%)	1 (7.7%)		3 (5.1%)	0 (0.0%)
	Sleep apnea			5 (6.2%)	1 (9.1%)		3 (7.1%)	0 (0.0%)		9 (15.3%)	1 (8.3%)
	Restless legs syndrome and periodic limb movement disorder			11 (13.8%)	0 (0.0%)		7 (16.7%)	2 (15.4%)		7 (11.9%)	1 (8.3%)
	Circadian rhythm sleep disorders			4 (5%)	1 (9.1%)		3 (7.1%)	0 (0.0%)		5 (8.5%)	1 (8.3%)
	Parasomnia			3 (3.8%)	1 (9.1%)		3 (7.1%)	0 (0.0%)		6 (10.2%)	1 (8.3%)
*Mental health*	Depression (RMT20)			9.5 (3.8)	8.5 (3.6)		12.0 (3.4)	12.2 (4.0)		12.3 (4.0)	12.4 (4.0)
	Major depressive disorder diagnosis			11 (13.8%)	1 (9.1%)		13 (31.0%)	8 (61.5%)		32 (54.2%)	5 (41.7%)
	Generalized anxiety (RMT20)			11.2 (3.7)	11.2 (4.5)		13.4 (2.9)	12.9 (3.8)		14.1 (3.0)	14.7 (2.5)
	Generalized anxiety disorder diagnosis			24 (30.0%)	2 (18.2%)		31 (73.8%)	10 (76.9%)		47 (79.7%)	11 (91.7%)
	Social anxiety (RMT20)			9.3 (3.8)	8.7 (3.5)		10.5 (3.9)	11.4 (3.7)		11.6 (4.4)	10.5 (4.3)
	Social anxiety diagnosis			9 (11.2%)	0 (0.0%)		13 (31.0%)	6 (46.2%)		26 (44.1%)	3 (25%)
	Panic (RMT20)			6.3 (3.1)	7.0 (3.6)		7.8 (3.5)	7.8 (3.5)		8.5 (3.6)	9.8 (3.1)
	Panic disorder diagnosis			6 (7.5%)	0 (0.0%)		8 (19.0%)	2 (15.4%)		12 (20.3%)	3 (25%)
	Trauma (RMT20)			7.0 (4.2)	8.0 (4.9)		9.3 (4.6)	8.0 (4.9)		10.7 (5.0)	10.1 (5.0)
	Post traumatic stress disorder diagnosis			9 (11.2%)	1 (9.1%)		10 (23.8%)	2 (15.4%)		35 (59.3%)	5 (41.7%)
	Borderline severity (BPD-C)			16.4 (6.0)	16.1 (6.0)		19.7 (5.7)	20.6 (6.8)		21.4 (7.4)	19.2 (7.4)
	Any diagnosis			36 (45.0%)	3 (27.3%)		39 (92.9%)	12 (92.3%)		59 (100%)	12 (100%)

^*^ Medication use refers to all medication prescribed by a healthcare provider, such as a general practitioner or psychiatrist, for treating insomnia or mental health issues (e.g., depression, anxiety). Note: Clinical diagnoses were assessed using the Mini International Neuropsychiatric Interview (M.I.N.I). Abbreviations: MHC: Mental Health Care; BPD-C: Ultra-Short Borderline Personality Disorder Checklist (range: 9–45); ISI: Insomnia Severity Index (range: 0–28); RMT20: Rapid Measurement-Toolkit 20 (subscale range: 4–20); SD: standard deviation

Of the 181 i-Sleep participants, 13 explicitly chose to discontinue the intervention: 5 (6.3%) from the pre-clinical group, 4 (9.5%) from general MHC group, and 4 (6.8%) from specialized MHC group. Exit interviews indicated that time constraints time constraints (5) were the most frequent cited reason, followed by health conditions (3). Other reasons included perceived irrelevance for menopausal women (1), excessive burden of the sleep diary (1), negative impacts on work and safety (1), and difficulties balancing iCBT-I with ongoing MHC therapy (2), with one of these instances being a decision made based on clinician advice. Andwhen asked if any changes could have helped them complete the program, nearly all participants (*n* = 12) felt that no additional modifications would have made a difference; only one participant suggested that clearer communication and expectation‑setting might have improved program adherence.

Additionally, some participants did not formally quit but failed to complete the intervention. Completers were defined as participants who completed all five sessions within the 8-week period. Non-completers were those who, for any reason, did not complete all five sessions within the 8-week period, even if they did not formally quit. After the 8-week period, attrition rates,reflecting both explicit quitters and non-completers, were 16.3% in the pre-clinical group, 19.1% in the general MHC group, and 30.5% in the specialized MHC group, resulting in an overall attrition rate of 21.6%. Completion status was monitored through the Netherlands Sleep Registry.

### RE-AIM: the effectiveness dimension

The Effectiveness dimension of the RE-AIM framework concerns the perceived effectiveness of the iCBT-I intervention, along with the satisfaction, views, and experiences of participants. Detailed results are presented in Table 4, 5, and 6. Table 4 provides quantitative insights, capturing participant satisfaction and perceptions regarding the various elements of the intervention, including its perceived effectiveness in addressing insomnia complaints and feasibility. Table 5 complements this with qualitative insights, summarizing participants’ feedback on their experiences. Lastly, Table 6 presents a mixed-methods overview of participant perspectives on the intervention’s guidance.

**Table 4:** Quantitative insights for satisfaction, views, and experiences with intervention elements (RE-AIM effectiveness dimension)

i-Sleep session and corresponding intervention elements		Pre-clinical group					General MHC group					Specialized MHC group					Overall			
		T1 *n* = 69			T2 *n* = 65		T1 *n* = 32			T2 *n* = 22		T1 *n* = 42			T2 *n* = 32		T1 *n* = 143			T2 *n* = 120
		*Feasibility T1; M (SD)*	*Effectiveness T1; M (SD)*	*Adherence T1; M (SD)*	*Adherence T2; M (SD)*		*Feasibility T1; M (SD)*	*Effectiveness T1; M (SD)*	*Adherence T1; M (SD)*	*Adherence T2; M (SD)*		*Feasibility T1; M (SD)*	*Effectiveness T1; M (SD)*	*Adherence T1; M (SD)*	*Adherence T2; M (SD)*		*Feasibility T1; M (SD)*	*Effectiveness T1; M (SD)*	*Adherence T1; M (SD)*	*Adherence T2; M (SD)*
Session 1																				
Bedroom surroundings		8.4 (2.3) NA: 23	6.6 (3.1) NA: 26	6.4 (3.7)	6.0 (3.2)		8.4 (2.3) NA: 7	7.1 (2.7) NA: 9	6.5 (3.7)	6.2 (2.5)		7.6 (2.6) NA: 8	6.4 (2.7) NA: 10	7.0 (3.0)	6.4 (2.8)		8.1 (2.4) NA: 38	6.6 (2.9) NA: 45	6.6 (3.5)	6.2 (2.9)
Evening ritual		7.8 (1.9) NA: 5	7.0 (2.5) NA: 7	7.5 (2.1)	6.8 (2.5)		7.3 (2.2) NA: 2	7.5 (1.9) NA: 5	7.5 (2.1)	6.6 (2.5)		7.5 (2.5) NA: 0	6.5 (3.1) NA: 2	7.7 (2.0)	6.5 (2.6)		7.6 (2.2) NA: 7	7.0 (2.6) NA: 14	7.5 (2.1)	6.7 (2.5)
Lifestyle adjustments		7.9 (2.0) NA: 5	6.4 (2.5) NA: 9	7.4 (2.1)	6.8 (2.0)		7.5 (1.8) NA: 3	6.8 (2.1) NA: 4	6.9 (2.3)	6.6 (2.4)		7.0 (2.2) NA: 1	6.2 (2.6) NA: 1	7.3 (1.8)	6.5 (2.3)		7.5(2.1) NA: 9	6.4 (2.4) NA: 14	7.2 (2.1)	6.7 (2.1)
Session 2																				
Fixed bedtimes		7.5 (2.2) NA: 2	7.6 (2.4) NA: 8	8.0 (2.1)	7.5 (2.0)		8.0 (2.0) NA: 1	7.9 (2.4) NA: 1	8.5 (2.0)	6.6 (2.3)		7.3 (2.6) NA: 1	7.4 (3.0) NA: 1	8.2 (2.2)	6.4 (2.6)		7.6 (2.3) NA: 4	7.6 (2.6) NA: 10	8.2 (2.1)	7.0 (2.3)
Sleep restriction		6.0 (2.9) NA: 4	7.3 (2.7) NA: 10	7.1 (3.0)	5.6 (2.8)		7.0 (2.7) NA: 2	6.9 (2.8) NA: 2	7.7 (2.9)	5.8 (2.9)		6.3 (3.0) NA: 1	6.9 (3.2) NA: 1	7.8 (2.5)	6.0 (2.8)		6.3 (2.9) NA: 7	7.1(2.9) NA: 13	7.4 (2.8)	5.8 (2.8)
Session 3																				
Relaxation exercises		7.2 (2.5) NA: 11	6.0 (2.7) NA: 11	6.0 (2.9)	5.0 (2.8)		7.7 (1.9) NA: 5	6.5 (2.0) NA: 6	6.2 (2.6)	4.9 (2.8)		6.8 (2.6) NA: 5	6.1 (2.9) NA: 5	5.5 (2.8)	4.9 (3.1)		7.2 (2.4) NA: 21	6.2 (2.6) NA: 22	5.9 (2.8)	5.0 (2.9)
Rumination (time)		6.8(2.6) NA: 18	6.0 (3.1) NA: 18	4.9 (3.2)	4.2 (2.7)		6.7 (2.2) NA: 9	5.7 (2.8) NA: 12	4.3 (2.6)	4.4 (2.5)		5.3 (2.9) NA: 9	4.6 (2.8) NA: 13	4.2 (2.5)	3.7 (2.6)		6.3 (2.7) NA: 36	5.5 (3.0) NA: 43	4.5 (2.9)	4.1 (2.7)
Rumination (other)		6.9 (2.5) NA: 8	6.0 (2.8) NA: 10	6.1 (2.8)	5.2 (2.7)		7.1 (2.3) NA: 3	5.9 (2.3) NA: 4	6.0 (2.5)	5.6 (2.7)		6.6 (2.7) NA: 5	6.3 (3.0) NA: 7	5.9 (3.0)	4.5 (2.9)		6.8 (2.5) NA: 16	6.1 (2.7) NA: 21	6.0 (2.8)	5.1 (2.8)
Session 4																				
Alternative thoughts		7.0 (2.5) NA: 9	6.0 (2.7) NA: 11	6.0 (2.8)	3.5 (2.5)		7.4(2.2) NA: 5	6.6 (2.4) NA: 7	5.9 (2.9)	4.3 (3.1)		6.3 (2.9) NA: 7	5.5 (3.2) NA: 11	5.5 (2.6)	3.6 (2.8)		6.9 (2.6) NA: 21	6.0 (2.8) NA: 29	5.8 (2.8)	3.7 (2.7)
Session 5																				
Future plan		-	6.4 (2.7) NA: 12	-	-		-	5.7 (2.4) NA: 7	-	-		-	5.6(2.5) NA: 8	-	-		-	6.0 (2.6) NA: 27	-	-

*Of note.* All items were assessed using a 10-point Likert scale (1 = not at all feasible, never performed, or not at all effective, and 10 = highly feasible, performed daily, or highly effective, with NA as not applicable) to evaluate feasibility, adherence and perceived effectiveness of the iCBT-I intervention. *Bedroom surroundings* refer to modifications in the sleep environment to promote better sleep quality, such as reducing, noise and light, temperature. *Evening ritual* involves establishing a calming pre-sleep routine, including activities like relaxation exercises. *Lifestyle adjustments* are changes aimed at improving sleep, such as limiting caffeine intake and ensuring regular physical activity. *Fixed bedtimes* refer to maintaining consistent sleep and wake times throughout the week to regulate the sleep cycle. *Sleep restriction* involves limiting time in bed to match the participant’s average total sleep time (TST), with gradual increases based on sleep efficiency (SE). *Relaxation exercises* include techniques like guided meditation and deep breathing to reduce arousal and facilitate sleep onset. *Rumination (time)* designates a specific period (e.g., 15 min) to process worries before bedtime, helping to reduce intrusive thoughts. *Rumination (other)* involve cognitive strategies aimed at managing unhelpful thoughts, such as focusing on staying awake or blocking repetitive thoughts. *Alternative thoughts* focus on challenging and modifying unhelpful thoughts related to sleep, replacing them with more adaptive and realistic perspectives. *Future plan* entails developing a personalized plan to prevent relapse into insomnia, summarizing the key strategies learned throughout the intervention

*Abbreviations.* NA: not applicable; MHC: mental health care; M: mean; SD: standard deviation

**Table 5:** Qualitative insights for satisfaction, views, and experiences with intervention elements (RE-AIM effectiveness dimension)

i-Sleep session and corresponding intervention elements		Participants experiences and quotes (*n* = 36) with MHC subgroups: pre-clinical (*n* = 11), general MHC (*n* = 13), specialized MHC (*n* = 12)		Therapists’ experiences and quotes (*n* = 9)	
Session 1					
Bedroom surroundings, evening ritual and lifestyle adjustments		Many participants were already familiar with and were practicing key sleep hygiene tips prior to the intervention. There was a consensus among participants that sleep hygiene is a *“good start*” of the intervention: *“I think it’s a good foundation. I also feel that I had already tried many of these things.”*– participant 3 (general MHC group). For some participants, ingrained lifestyle habits, such as smoking/smart phone use, proved to be especially challenging to address.		Therapists recognized sleep hygiene as fundamental, noting it serves as an essential starting point. However, they stated that additional guidance beyond the initial introduction between therapist and participant may not be necessary. One therapist remarked: *“Sleep hygiene is often a bit of an open door. The extra addition of guidance is good for initial introduction and such*,* but intensive steering is usually not needed.” - therapist 1.*	
Session 2					
Fixed bedtimes and sleep restriction		Many participants found sleep restriction to be highly beneficial. As one participant summarized, “*The most valuable and also the most challenging*.”– participant 4 (general MHC group). Remarkably, nearly all participants were unfamiliar with this approach prior to the intervention. Most participants reported significant tiredness as a side effect. One participant noted, “*It made me tired*,* yes. I also encountered problems with planning*,* which started to hinder my functioning. At that moment*,* it became practically impossible for me to manage*.”– participant 5 (specialized MHC group). Another participant described their experience as, “*I was utterly exhausted.*”– participant 6 (specialized MHC group).		Therapists highlighted their perceived effectiveness of sleep restriction, noting that “*I think it’s the most important component of i-Sleep because it works.”*– therapist 2. Therapists also emphasized the value of guidance: “*With sleep restriction*,* guidance is particularly valuable. It helps you assist someone more effectively and challenge them to spend less time in bed*,* as people often opt for the easiest solution. Therefore*,* guidance is essential in this context.”*– therapist 1.	
Session 3					
Relaxation exercises and rumination (time and other)		For most participants, the relaxation exercises proved useful and effective. There were some criticisms on the style of the audio fragments used in these exercises. Within the specialized MHC groups, some participants reported that the relaxation exercises had an adverse effect. One participant remarked, *"I really couldn’t manage that*,* because*,* you see*,* I also have traumas*,* and consciously engaging with relaxation and being in tune with my body that way actually triggered a lot of anxiety for me. So*,* it made that session very challenging for me*,* and it’s actually one of the reasons why I ultimately didn’t complete the entire module. Because I noticed it brings up a lot of things that I can’t handle right now.”*– participant 7 (specialized MHC group).		Therapists noted that creating detailed plans with participants, outlining specific yet simple activities designed to promote relaxation, was generally well-received by participants. However, some therapists expressed that not all participants fully engaged with or understood the importance of these activities. As one therapist observed, *“Some don’t understand the importance of taking time for themselves to relax.”* – Therapist 5.	
Session 4					
Alternative thoughts		Participants reported increased awareness of their negative thought patterns and their impact on sleep. Yet many found it challenging to consistently alter these thoughts. As one participant explained, *“Yes*,* I found that quite difficult. I remember there were a few thoughts that I could reframe*,* but I often struggled to come up with alternative thoughts for certain ideas. It was challenging for me to handle that.”– participant 8 (specialized MHC group).* The relevance of addressing negative thoughts varied among participants. Some found the intervention element highly beneficial, while others perceived it as less applicable, particularly if they did not experience any significant negative thoughts about sleep.		Therapists noted diverse responses to cognitive restructuring and stressed that a single session may not be adequate for some participants: *“if people have more severe [mental health] problems*,* addressing the cognitive aspect in just one session isn’t really sufficient. Often*,* they not only struggle with sleep but also with other issues*,* making it feel somewhat awkward to tackle it that way. In such cases*,* I think face-to-face treatment would be preferable. For certain target groups*,* you could use this approach*,* but in a limited way.“- therapist 3.* Therapist also noticed that: *“For topics like cognitive challenges*,* some people may easily overlook them when filling out the session. They might just provide a random thought or claim they don’t have any problems*,* indicating they haven’t fully understood the assignment. I’ve noticed that without our presence*,* they might not even engage with these aspects.” - therapist 3*	
Session 5					
Future plan		Participants appreciated the opportunity to review and focus on the strategies that were effective for them, noting, “*It’s helpful to review and maintain the points that worked.*”– participant 2 (pre-clinical group). They also recognized that the future-plan session serves an important role, as expressed by another participant: “*The real work starts after finishing the program*,* [the future plan] is a prompt to keep applying what you’ve learned and not revert to old patterns.”*– participant 9 (pre-clinical group). Some participants suggested that the future plan session could be more compact and presented in a bullet-point style for easier reference.		No remarks.	

*Of note. Bedroom surroundings* refer to modifications in the sleep environment to promote better sleep quality, such as reducing, noise and light, temperature. *Evening ritual* involves establishing a calming pre-sleep routine, including activities like relaxation exercises. *Lifestyle adjustments* are changes aimed at improving sleep, such as limiting caffeine intake and ensuring regular physical activity. *Fixed bedtimes* refer to maintaining consistent sleep and wake times throughout the week to regulate the sleep cycle. *Sleep restriction* involves limiting time in bed to match the participant’s average total sleep time (TST), with gradual increases based on sleep efficiency (SE). *Relaxation exercises* include techniques like guided meditation and deep breathing to reduce arousal and facilitate sleep onset. *Rumination (time)* designates a specific period (e.g., 15 min) to process worries before bedtime, helping to reduce intrusive thoughts. *Rumination (other)* involve cognitive strategies aimed at managing unhelpful thoughts, such as focusing on staying awake or blocking repetitive thoughts. *Alternative thoughts* focus on challenging and modifying unhelpful thoughts related to sleep, replacing them with more adaptive and realistic perspectives. *Future plan* entails developing a personalized plan to prevent relapse into insomnia, summarizing the key strategies learned throughout the intervention

*Abbreviations.* MHC: mental health care

**Table 6:** Mixed methods result for satisfaction, views, and experiences post intervention (T1) of therapist guidance during intervention (RE-AIM effectiveness dimension)

	*Quantitative insights*				*Qualitative insights*	
	Mean (SD) or n (%)					
*Elements of therapist guidance*	**Pre-clinical group** (*n* = 69)	**General MHC group ** (*n* = 32)	**Specialized MHC** **group** (*n* = 42)	**Overall** (*n* = 143)	**Participants experiences and quotes (***n* **= 36) with MHC subgroups: pre-clinical (*n*** **= 11)**,** general MHC (*n*** **= 13)**,** specialized MHC (*n*** **= 12)**	**Therapists’ experiences and quotes** **(*n*** **= 9)**
Overall satisfaction guidance	7.9 (1.7)	8.3 (1.7)	7.7 (1.5)	8.0 (1.7)	Participants were generally very satisfied with the therapist’s guidance, finding it important for understanding the intervention, helping to apply it to their personal circumstances and helping with adherence. One participant noted, *“I really think that having someone there to watch over you*,* answer your questions*,* and respond promptly and effectively is a fundamentally important part of the program. It might sound exaggerated*,* but knowing someone is keeping an eye on you and is there for you is very valuable. People who sleep poorly don’t always function at their best during the day*,* whether at work or in their personal lives. It’s just very helpful to have someone on the sidelines watching over you”*– participant 9 (pre-clinical group). Opinions on feedback varied regarding frequency, number of sessions, and delivery methods. Some participants felt that less frequent guidance would have sufficed, while others desired more regular interactions. There was also interest in incorporating optional contact moments at the end of the intervention to provide additional support. Interviews revealed a wish for more timely and personalized guidance, potentially through different feedback delivery formats. One participant suggested, *“Maybe having real conversations could also be helpful. It could make things a bit more personal*,* because*,* while I understand there’s a certain approach in place*,* having one-on-one conversations allows for immediate feedback”*– participant 10 (specialized MHC group). Technical issues, delayed feedback and slow responses to questions, were also noted. Additionally, the specialized MHC group noted the importance of improved coordination between the MHC practitioner and the iCBT-I therapist to ensure their treatments are aligned and supportive.	Therapists perceived their guidance to be important for motivating participants and ensuring adherence to the iCBT-I exercises, though the need for such guidance varied by session. As one therapist noted: *“I believe that even though the guidance is digital*,* it helps prevent participants from dropping out of the treatment. There’s a sort of commitment*,* even if it’s digital*,* creating a kind of agreement to tackle the issues. This helps ensure that participants continue with the intervention. Without regular communication with a therapist*,* I believe they more likely to quit.”*– therapist 3. Building a strong therapist-patient connection was sometimes challenging as some participants provided brief answers with minimal reasoning. Therapists also highlighted the need to improve data accuracy. Errors in reported bedtimes occasionally led to miscalculations, requiring manual re-calculations and adjustments to guidance. Opinions on the format of feedback delivery varied. Some therapists suggested that while the online module is beneficial, face-to-face treatment might be preferable for complex cases, as it offers more comprehensive care. Others emphasized the value of opt-in guidance, with one therapist noting, *“If you have a really challenging participant who struggles with the assignments or doesn’t fully understand something*,* a phone call could be helpful. You can explain things much more quickly and clearly*,* but this isn’t necessary for every participant, it’s more of a case-by-case need.*”– therapist 4. Another therapist highlighted the benefits of an automated version, stating: *“A more automated version can be advantageous because it clearly focuses on sleep-related skills without being overly complex*,* making it useful for all”–* Therapist 5.
Sufficiency of *personalized* guidance (n, %)	60 (87.0%)	29 (90.6%)	32 (76.2%)	121 (84.6%)		
Therapist gave good advice	7.8 (2.4)	8.3 (1.8)	7.7 (1.7)	7.8 (2.1)		
Therapist considered personal circumstances	7.5 (2.8)	8.5 (1.9)	7.5 (2.2)	7.7(2.5)		
Therapist availability	8.0 (2.2)	8.3 (2.0)	7.9 (2.2)	8.0 (2.1)		
Therapist provided options	7.9 (2.3)	8.3 (1.8)	7.5 (2.1)	7.9 (2.1)		
Therapist guided the progress	8.0 (2.1)	8.2 (1.9)	7.8 (2.1)	8.0 (2.0)		
Therapist motivated completion	7.6 (2.7)	8.0 (2.3)	7.9 (1.8)	7.8 (2.4)		
Therapist instilled confidence	7.0 (3.1)	7.8 (1.9)	7.0 (2.3)	7.2 (2.7)		
Therapist took time	7.8 (2.3)	8.5 (1.9)	7.7 (2.2)	7.9 (2.2)		

*Note*: All items, except for the “sufficiency of personalized guidance” variable (measured as a yes/no variable), were evaluated using a 10-point Likert scale, where 1 indicates “completely disagree” and 10 indicates “completely agree.”

*Abbreviations.* MHC: mental health care; M: mean; SD: standard deviation

#### Perceived effectiveness of the iCBT-I intervention: participant satisfaction, views, and experiences

Participants’ satisfaction with the iCBT-I intervention at T1 was positive across all groups. In the pre-clinical group (*n* = 69), the mean satisfaction rating was 7.1 (SD = 1.6). The general MHC group (*n* = 32) reported a mean rating of 7.3 (SD = 1.4) and the specialized MHC group (*n* = 42) had a mean rating of 7.1 (SD = 1.7). Overall, 93.0% of participants found the content, advice, and homework assignments provided in the iCBT-I intervention to be sufficiently clear and understandable. Specifically, the rates were 94.2% for the pre-clinical group, 93.8% for the general MHC group, and 90.5% for the specialized MHC group. For more detailed information on the perceived effectiveness and feasibility of each intervention element, see Tables 4 and 5.

Participants interviews provided valuable insights into the perceived effectiveness of the iCBT-I intervention on sleep, self-awareness, lifestyle, and mental health. Many participants reported significant improvements in sleep onset, duration, and quality, including falling asleep more quickly, waking up less frequently during the night, and experiencing an overall improvement in sleep. These enhancements often led to increased daytime energy. Additionally, some participants noted positive lifestyle changes, such as enhanced social interactions and reduced alcohol consumption. Regardless, some participants did not perceive any effect on their sleep. One participant shared, “*I don’t really notice a difference in how well I sleep*”– participant 6 (pre-clinical group). Persistent issues, such as nighttime awakenings and nightmares, were also reported: “*No one can do anything about my nightmares. So if I have disturbing thoughts or I’m very stressed or worried about something before I go to sleep*,* I can already tell myself: it’s going to be a hellish night where I will hardly sleep or not sleep at all.*’”– participant 11 (specialized MHC group). Increased self-awareness was a valued outcome for most interviewed participants. Tools like sleep diaries were particularly effective in fostering awareness and a deeper understanding of sleep patterns and behaviors. As one participant observed, “*The module provided a lot of insight into how I actually deal with sleeping*,* going to bed*,* and all the surrounding factors. And it was clear that there was room for improvement”*– participant 9 (pre-clinical group).

The perceived effect of the iCBT-I intervention on mental health and emotional well-being varied among the interviewed participants. Several participants reported improvements in their emotional well-being because of better sleep. For instance, one participant from the pre-clinical group reflected on the broader benefits, stating, *“I now fall back asleep more easily*,* which makes me feel better overall.”* - participant 12 (pre-clinical group). A participant from the general MHC group described a notable reduction in anxiety due to improved sleep: “*Because of the better sleep*,* my anxiety has decreased significantly. Feeling more rested has improved my overall well-being and resilience. I can handle more without becoming overtired.”*– participant 13 (general MHC group). However, not all experiences were entirely positive. Some participants reported none or negative effects on their emotional well-being and mental health. These adverse experiences were often linked to specific components of the iCBT-I intervention, such as sleep restriction or relaxation exercises. A participant from the pre-clinical group shared, “*The restriction had a very negative impact on my mood. It was so bad that I became really depressed again and thought*,* ‘If I keep going with this*,* I’m going to fall too far behind*,* and I don’t know if that’s good for me.*‘”– participant 14 (pre-clinical group). Conversely, a participant from the specialized MHC group described how these side effects eventually gave way to better sleep and greater emotional stability: *“At the beginning*,* with the sleep restriction*,* it was quite tough at times. When I was really tired*,* I would become more easily anxious. But now*,* that’s gone*,* and I think that because I’m sleeping better*,* everything is overall better*,* including feeling less anxious.*”– participant 8 (specialized MHC group).

### RE-AIM: the adoption dimension

The Adoption dimension of the RE-AIM framework concerns therapist demographics and their reasons for engaging with, continuing, or opting out of the intervention.

#### Therapist demographics and factors influencing their intervention engagement

A total of 15 therapists guided participants through the i-Sleep program. The therapists had an average age of 30.1 years (range: 24–48), with 13 females and 2 males. Their roles included research assistants, PhD students, and MSc students, all with a background in (neuro)psychology, and two licensed psychologists specializing in CBT-I. Most had limited clinical experience in mental health care settings (average 0.8 years), although five therapists had extensive experience with the i-Sleep program in prior research contexts. In contrast, the two licensed psychologists had more extensive clinical experience. Before guiding i-Sleep participants, therapists rated their beliefs about the effectiveness of addressing sleep problems on a 5-point Likert scale. Results showed strong agreement that discussing sleep issues improves daily functioning (M = 4.8, SD = 0.6) and reflected high motivation to address these problems (M = 4.8, SD = 0.4). Therapist motivations for participating in the study varied: some aimed to enhance their understanding of sleep disorders, others sought to improve treatment outcomes for patients with significant sleep disturbances, and some were driven by a personal interest in contributing to scientific research.

### RE-AIM: the implementation dimension

The Implementation dimension assesses treatment adherence among participants, protocol adherence by therapists, use of training and support, and therapists’ satisfaction with the intervention and provided support.

#### Intervention adherence among participants

Interviews with the participants revealed that continued participation was primarily driven by personal commitment, perceived benefits, therapist support, and a sense of duty towards contributing to the research. As one participant from the general MHC group noted, *“Well*,* I like to finish what I start. Also*,* I felt that the person answering my questions was very dedicated and thorough. I thought*,* ‘This person is putting a lot of effort into helping me*,*’ so I wanted to reciprocate that commitment. Moreover*,* it was a real issue that I genuinely wanted to resolve. If you’re going to do something*,* you need to give it your all.”*– participant 4 (general MHC group). Table 4 provides an overview of adherence rates at T1 to the specific intervention elements.

#### Therapist adherence to the intervention protocol, use of training and support, and satisfaction with the iCBT-I intervention

Therapists appreciated the guidance process, despite occasional challenges in building therapist-patient connections through digital platform. They identified the need for direct participant support in areas such as sleep restriction and cognitive restructuring. Experiences with the feedback delivery method varied, with some therapists preferring face-to-face interactions for complex cases, while others favored the efficiency of automated guidance. Overall, they agreed that structured support was essential for maintaining participant motivation and adherence. On average, therapists provided 31 min (SD = 6.6) of guidance per session, totaling to 2.6 h (SD = 0.6) of guidance per participant across the five-session intervention. In comparison, previous i-Sleep studies reported guidance durations of 5–20 min per session by nurses in GP practices [21] and approximately 40 min per session by psychology students [42]. Therapists also sent an average of 2.5 reminders (SD = 3.0) per participant, in line with a standardized reminder protocol applied across all groups. Across care levels, completers in the pre-clinical group received an average of 2.6 h (SD = 0.5) of guidance, the general MHC group received 2.5 h (SD = 0.5), and the specialized MHC group received 2.5 h (SD = 0.6) of guidance. Therapist engagement was self-reported in logbooks.

Therapists reported positive experiences with the i-Sleep training and weekly case discussion meetings. Therapists rated their confidence and readiness in providing the i-Sleep intervention post-training as 8.1 (SD = 1.0) on a 10-point Likert scale. Qualitative insights from focus groups confirmed high satisfaction but also highlighted areas for improvement. Suggestions included tailoring training content to therapists’ prior experience, providing concise materials for experienced practitioners, and focusing on online facilitation techniques for those new to digital interventions. Therapists valued the support and collaborative learning fostered by the weekly case discussion meetings. They appreciated consulting an experienced CBT-I professional outside these meetings for timely feedback on participant progress. One therapist noted: “*I personally find the case discussion meetings very enjoyable. [.] Additionally*,* having a regular weekly time to ask questions is very valuable. I’ve noticed that I don’t often have specific*,* concrete questions that need immediate answers*,* as I can usually address those via email. However*,* during these case discussion meetings*,* discussing my participants often brings up challenges I hadn’t considered before. I appreciate receiving tips and suggestions from others*,* as everyone brings a unique perspective*,* which often proves to be very helpful”–* therapist 3. However, opinions varied regarding the frequency of these meetings, with some finding them excessive, especially when discussions included participants with no significant updates. Concerns were also raised about the mandatory nature of the meetings, with some therapists questioning their practicality and efficiency in a real-world clinical setting.

### RE-AIM: the maintenance dimension

The Maintenance dimension evaluates the long-term helpfulness and continued use of the intervention elements by participants. It also examines therapists’ intentions regarding ongoing use of the intervention.

#### Participant insights on long-term outcomes and continued utilization of the iCBT-I intervention

At T1 (n = 143), 89.4% of the i-Sleep participants expressed a willingness to recommend the iCBT-I intervention, with recommendation rates of 88.4%% for the pre-clinical group, 87.1% for the general MHC group, and 92.9%% for the specialized MHC group. Despite these high recommendation rates, interviews revealed more nuanced perspectives. Some participants emphasized selective recommendations, preferring a more interactive format or considering individual motivation levels. They also advised caution in recommending the intervention for individuals with severe trauma-related nightmares, burnout, chronic pain, menopause-related issues, the elderly, or those with extensive prior mental health treatments. As one participant noted, *“It depends a bit on how severe it is. I think it’s more suited for younger people with sleep issues. For older adults*,* you would need to create something more specific.”*– participant 15 (pre-clinical group). At T2 (n = 120), six months post-intervention, adherence to various components of the iCBT-I intervention was evaluated. Table 4 shows that the highest adherence was for maintaining a fixed sleep schedule (m = 7.0, SD = 2.3), while adherence was lower for rumination exercises (worry) (m = 4.1, SD = 2.7) and correcting negative sleep beliefs (m = 3.7, SD = 2.7).

Interviews highlighted effective elements of the intervention that participants would consider reusing for recurring insomnia symptoms. Many preferred to selectively apply specific exercises, such as sleep restriction (including fixed bedtimes) and relaxation techniques, rather than re-engage with the entire module. Participants also emphasized the importance of using a sleep diary to monitor sleep patterns. One participant remarked, *“I think what i-Sleep has really given me*,* apart from the overall experience*,* is that I now have more tools to use when I start having trouble sleeping again. I can particularly rely on sleep restriction*,* which I find very helpful. For instance*,* when I notice I’m not sleeping well for a few nights*,* I get stricter with myself*,* and that helps. By increasing my sleep pressure again*,* I avoid lying awake at night.”*– participant 16 (specialized MHC group). Questionnaire responses at T2 revealed that 24.6% of the pre-clinical group, 27.3% of the general MHC group, and 33.3% of the specialized MHC group sought additional methods to address sleep complaints after the intervention. Many of these methods, such as reducing alcohol and caffeine intake and practicing mindfulness, were already incorporated into the iCBT-I intervention. However, participants also explored additional strategies beyond the intervention, including biofeedback, increased exposure to natural daylight, and the use of alternative sleep aids or acupuncture.

#### Therapist insights on ongoing use and integration of the iCBT-I intervention

Although some therapists had limited clinical experience, they recognized the potential of the guided iCBT-I intervention for long-term use and integration into routine practice. Several strategies were proposed to enhance training and case discussion meetings for real-world settings. For example, transitioning from weekly to bi-weekly case discussions was suggested along with enhancements to training content that tailor materials to therapists’ prior experience. Specifically, improvements were recommended in structured written guidance, digital rapport-building techniques, and working with a more structured delivery format for those less familiar with delivering digital interventions.

### Recommendations for improvement of the intervention and implementation support beyond the research setting

The following identified recommendations address content, flexibility and personalization in guidance, accessibility and usability, and the use of sleep as an accessible gateway to mental health care.

#### Content

Participants emphasized the need for integrating comprehensive information on sleep medications alongside sleep restriction protocols to better understand their impact on sleep patterns. Therapists supported this suggestion, noting, “*I noticed that there isn’t a clear section discussing medication or the types of medication participants might be using. Some people*,* for example*,* might self-prescribe over-the-counter melatonin.”*– therapist 4. Additionally, participants expressed a desire for clearer introductions to the intervention, including more detailed expectations to better manage their anticipation. One participant noted, “*I was so exhausted from the sleep restriction that*,* if I had known how challenging it would be*,* I would have chosen a different week*,* maybe a vacation week.”*– participant 17 (general MHC group). Feedback on the experience-based case vignettes of ‘example’ participants with comorbid psychiatric symptoms was mixed. Many participants either did not engage with these vignettes or found them ineffective, while others appreciated them for making them feel understood. The consensus was to retain the vignettes in the program but enhance their attractiveness and relevance, potentially through video formats or more engaging presentations. Participants further suggested incorporating more self-reflection moments within the intervention to monitor progress and assess effectiveness. To prevent monotony, diversifying relaxation techniques was advised, as many preferred alternative tools, such as those with better audio quality or more varied exercises. Addressing specific subjects, such as (PTSD-related) nightmares and sleep complaints during menopause, was also suggested to better tailor the intervention to diverse needs.

#### Flexibility and personalization in guidance

Both participants and therapists expressed a need for greater flexibility in the duration and format of guidance within the iCBT-I intervention. Some participants suggested adding optional check-in sessions to sustain engagement, with preferences divided between structured weekly feedback and (optional) on-demand support. Therapists shared these concerns, noting that the current five-week timeframe might be insufficient for *all* participants to fully absorb and apply the skills taught. Incorporating optional check-ins and varied feedback methods could enhance engagement and satisfaction. Furthermore, both groups emphasized the importance of personalized support. Some participants sought more individualized guidance, while therapists suggested improving personal connections within the online module to strengthen participant commitment. Proposed enhancements included opt-in phone interactions and simple features, such as displaying a therapist’s photo in the account or chat for feedback. Although face-to-face or phone/video interactions could provide more tailored assistance, therapists noted that increased automation might benefit participants who prefer to progress independently.

#### Accessibility and usability

To enhance the intervention’s accessibility, participants recommended several key improvements. They suggested expanding availability across various platforms and developing a dedicated intervention app with features beyond the current sleep diary function. Both participants and therapists emphasized the importance of addressing IT issues by making the sleep diary more intuitive and providing clearer insights. Participants also expressed a desire to revisit and review previous session responses, as only therapist feedback is currently accessible. Additionally, offering a non-digital version of the intervention for those without digital access or skills could further improve accessibility. Incorporating visual aids to clarify key concepts was also recommended to enhance comprehension.

#### Sleep as an accessible gateway to mental health care

Some participants perceived sleep treatment as a less intimidating entry point for addressing mental health issues. One participant remarked, *“Well*,* as soon as you have sleep problems*,* I would advise this as the first step. There’s not such a high barrier of ‘oh*,* then you have to go to therapy’ or something like that”* (participant 14– pre-clinical group). This perspective suggests that interventions like iCBT-I may lower perceived barriers to seeking mental health support, highlighting a potential focus for implementation efforts.

## Discussion

This study evaluated the implementation of therapist-guided digital cognitive behavioral therapy for insomnia (iCBT-I) in individuals with mental health issues, regardless of whether they were unattended or referred to general or specialized mental health care. The evaluation was structured according to the RE-AIM framework, focusing on its reach, effectiveness, adoption, implementation, and maintenance. Ongoing efforts, such as those led by the European Insomnia Network [43], aim to enhance the accessibility and quality of CBT-I. This process evaluation contributes to these efforts by presenting both quantitative metrics and detailed qualitative insights obtained from iCBT-I participants and therapists. These findings not only provide recommendations for optimizing the iCBT-I intervention in mental health care settings but also offer a comprehensive understanding of the implementation challenges and opportunities associated with integrating iCBT-I into clinical practice.

### Attrition and delivery of therapist guided iCBT-I

Participants in our study highlighted the importance of therapist guidance within the iCBT-I intervention, with most reporting that it motivated them and encouraged continued engagement. This is further supported by our attrition rates which were notably lower than those seen in fully automated digital interventions [44–47]. Attrition rates were 16.3% in the pre-clinical group, 19.1% in the general MHC group, and 30.5% in the specialized MHC group, resulting in an overall rate of 21.6%. In contrast, larger studies on fully automated digital CBT-I have reported much higher non-completion rates. For example, two out of five participants failed to complete four or more sessions [44] and that three out of five did not finish the six-week intervention [45, 46]. Additionally, only 331 out of 1,891 participants in a third study completed the full course, i.e. non-completion in more than four out of five [47]. Notably, these studies did not specifically target individuals with mental health issues, who are known to be more likely to drop out of treatment with CBT targeting insomnia [48, 49]. The variation in attrition rates across care settings in our study do underscore the need for investigation into which groups are most vulnerable to discontinuation. Exploring different formats of therapist guidance is thus essential for enhancing feasibility, retention, and participant satisfaction, particularly for those at greater risk of dropout. Notably, forthcoming results from the *‘Better nights, better days?’* study in a borderline personality disorder population [25], which includes three additional 30-minute video call appointments during i-Sleep, may reveal how such complementary guidance impacts attrition rates.

While participants appreciated the written digital guidance provided by therapists, some expressed a desire for greater flexibility and personalization in both the content and type of support. This demand for tailoring is also observed in fully automatic interventions [50]. For example, during the initial session focusing on sleep hygiene, minimal guidance was required, as many participants had already addressed these topics. This was reflected in high ‘not applicable’ rates and feedback from therapists indicating a limited need for intervention. These findings suggests that certain aspects of iCBT-I could be effectively automated. Conversely, more complex components, such as cognitive restructuring, may benefit from additional opt-in guidance to offer a more individualized approach when necessary.

### Guided iCBT-I: what works for whom?

The intervention was generally well received, though the variability in participant experiences highlights the limitations of a one-size-fits-all approach. Participants in the general mental health care (MHC) group rated elements like fixed bedtimes, sleep restriction, and relaxation exercises as more feasible and effective compared the other care levels. The specialized MHC group reported lower feasibility and effectiveness, for elements such as rumination, alternative thoughts, and relaxation exercises. Some participants in the specialized group found relaxation exercises aversive, a finding consistent with research showing that trauma-informed mindfulness can benefit individuals with PTSD but may also cause distress in certain cases [51]. Adherence scores at T1 and T2 were similar across groups, although adherence to the “alternative thoughts” component at T2 was notably low, suggesting difficulties with long-term integration or resolution of these issues at follow-up. Moreover, participants in the specialized group expressed a desire for better integration of iCBT-I into their broader mental health care treatment plans, highlighting a need for coordinated care. Insights from therapists delivering both i-Sleep and the upcoming MHC treatments could offer valuable perspectives for refining future implementation strategies [52].

### Possible adjustments in iCBT-I

It seems clear that a one-size-fits-all approach may not be optimal for every individual. Modifying iCBT-I to better address specific needs could improve its effectiveness and uptake. Incorporating optional modules to target specific issues, such as sleep complaints in menopausal women or PTSD-related nightmares, was suggested by participants in this study. Context-, condition-, and individual-focused adaptations to CBT-I have been made, however, further research is needed to identify *when* and *how* to implement these adaptations in iCBT-I and to evaluate their benefits [53]. For instance, integrating nightmare therapy into CBT-I for PTSD has shown promise in reducing sleep disturbances and improving sleep quality [54]. CBT-I has also been adapted transdiagnostically for individuals with severe mental disorders during inpatient psychiatric care [55, 56].

Our qualitative data further revealed diverse experiences with sleep restriction, with some participants finding it particularly burdensome and emotionally distressing. Although the challenging nature of sleep restriction is well-documented, it is widely regarded as one of the most effective components of CBT-I [57]. This underscores the importance of strategies that not only motivate patients but also manage their expectations to ensure continued engagement. A recent study found no significant differences between sleep restriction and sleep compression on objective sleep outcomes after 10 weeks, suggesting that sleep compression may provide a gentler alternative [58]. Notably, one participant in our study reported an increase in anxiety during the initial phase of sleep restriction, followed by subsequent improvements. This raises critical questions about whether such exacerbations are temporary and whether alternative or supplementary forms of therapist guidance could better address these challenges.

### Strengths and limitations

A key strength of this study is its inclusion of participants exhibiting clinically relevant symptoms across a range of emotional distress disorders and all levels of mental health care, from pre-clinical stages to those awaiting specialized treatment. Furthermore, the study employs an effectiveness-implementation Hybrid Type 2 design and utilizes a mixed-methods approach with lenient inclusion criteria. However, several limitations must be acknowledged. Potential biases in participant selection and therapist representation may have impacted the results. Recruitment strategies tailored to the effectiveness trial may have relied on explicit offering of CBT-I and pragmatic use of the waiting period, raising questions about uptake in different conditions. Additionally, given our hybrid design and the limited clinical experience of most therapists, future research should explore broader clinical adoption, including insights from more seasoned clinicians, to evaluate efficient training methods, provider bandwidth, and optimal integration into healthcare workflows. These efforts are essential for enhancing the scalability and real-world implementation of iCBT-I. It is also important to note that while quantitative data collection for both the general and specialized mental health care groups is still ongoing, the decision to publish at this stage is based on achieving qualitative data saturation and the prioritization of publishing the process evaluation before efficacy results, in line with recommendations of UK MRC guidelines [59]. Lastly, the dose-response relationship, part of the RE-AIM framework, is not addressed in this paper.

## Conclusion

Taken together, people with mental health issues experienced a therapist-guided digital CBT-I as effective and feasible, an impression also shared by the therapists. However, there remains room for optimization. We identified two main recommendations: (1) guidance and content may need to be adjusted based on what a patient needs most. It is thus important to consider specific needs, incorporate ongoing assessments, and explore alternative or supplementary guidance when necessary. This could either be in type of delivery mode (e.g. add telephone or use automated text for simpler parts) or in the amount of support (intensifying if necessary). (2) Patient characteristics seem to matter; iCBT-I may not be suitable for everyone. While it may be highly effective for some, others may experience less benefit or even negative outcomes. These recommendations align with the stepped care model, which advocates for delivering interventions that match the specific needs of patients at different levels of care [60]. The obvious challenge lies in determining who needs what type of guidance and identifying the individuals best suited for iCBT-I. Additionally, our study indicates that some participants sought further methods to improve their sleep post-intervention, suggesting that iCBT-I might not be a definitive solution for everyone. This aligns with existing literature indicating that while (i)CBT-I effectively reduces insomnia severity, quite some individuals do not achieve remission [61].

In conclusion, our findings underscore the potential of therapist-guided iCBT-I to improve sleep in individuals with mental health issues across various levels of care. While universal implementation in mental health care may offer substantial benefits, incorporating opt-in adaptations and enhancing flexibility in guidance frequency and format could further optimize the intervention, better addressing individual needs and mitigating non-compliance. Ongoing research, together with our forthcoming efficacy results [26], will aid in better understanding the full impact of applying therapist-guided iCBT-I in mental health care and further refining implementation strategies.

## Electronic supplementary material

Below is the link to the electronic supplementary material.

Supplementary Material 1

## Data Availability

Anonymised data of participants that approved of use for other studies are available upon request including a research question and data-analysis plan at info@slaapregister.nl.

## Abbreviations

AUDIT: Alcohol use disorders identification test
BPD: Borderline personality disorder
BPD-C: Ultra-short borderline personality disorder checklist
CBT-I: Cognitive behavioral therapy for insomnia
DUDIT: Drugs use disorders identification test
GAD: Generalized anxiety disorder
GP: General practitioner
ISI: Insomnia severity index
iCBT-I: Digital cognitive behavioral therapy for insomnia
M: Mean
MDD: Major depressive disorder
MHC: Mental health care
M.I.N.I: Mini international neuropsychiatric interview
NA: Not applicable
OCD: Obsessive-compulsive disorder
PD: Panic disorder
PTSD: Post-traumatic stress disorder
Q: Questionnaire
RCT: Randomized controlled trial
RMT20: Rapid measurement-toolkit 20
SAD: Social anxiety disorder
SD: Standard deviation
SE: Sleep efficiency
TST: Total sleep time
