# Supplementary material for: Applying therapist-guided digital cognitive behavioral therapy for insomnia in psychiatry: a mixed-methods process evaluation
Source: BMC Psychiatry. 2025 Apr 28;25:428. doi: 10.1186/s12888-025-06824-1 (PMC12039299; doi:10.1186/s12888-025-06824-1)
Supplement: Supplementary file 1 — Supplementary Material 1 [file 12888_2025_6824_MOESM1_ESM.docx]

**Appendix 0 - Flowchart of effectiveness-implementation hybrid type 2 trial design**

**
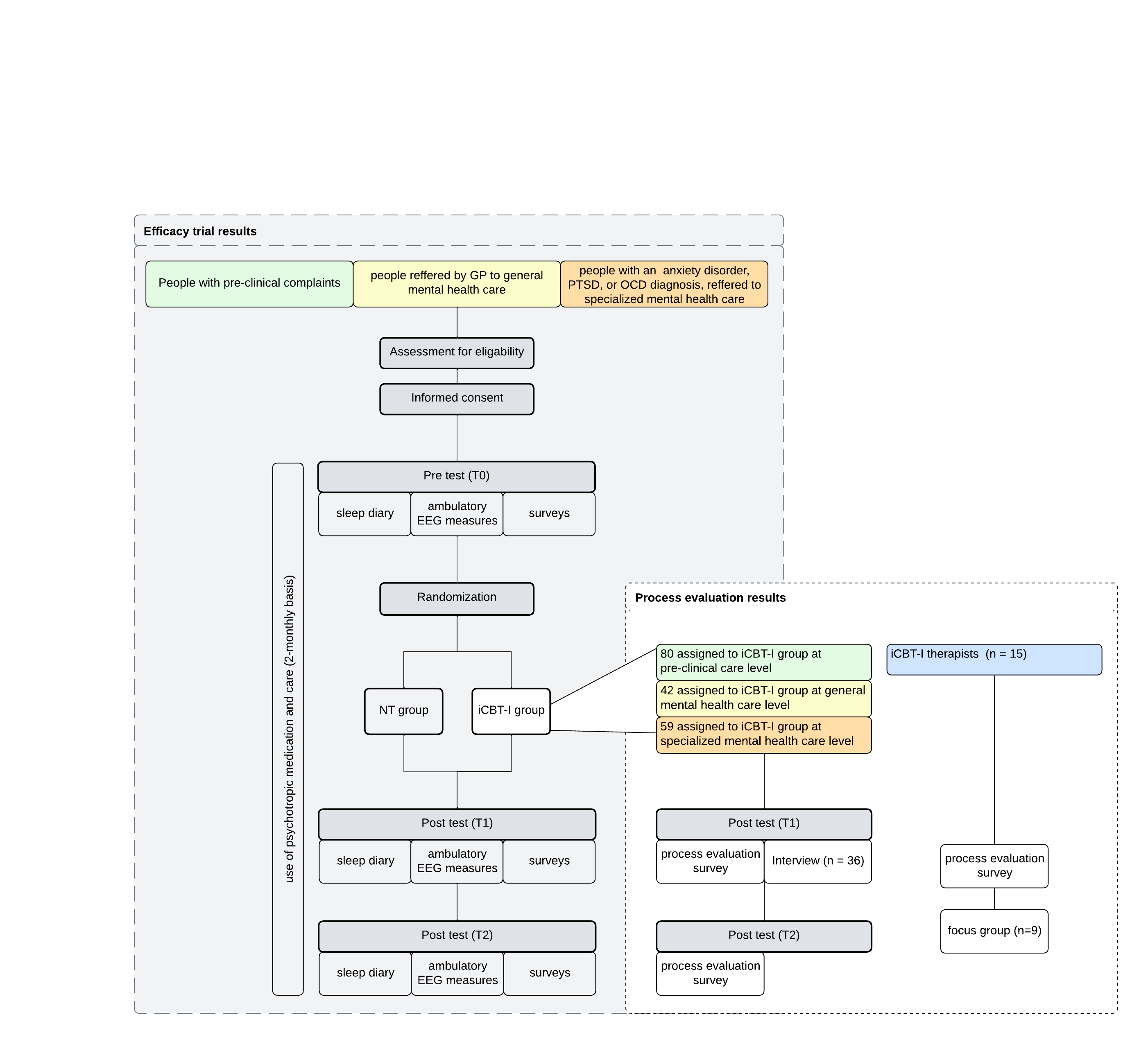
**

**Figure S1.** Flowchart of effectiveness-implementation hybrid type 2 trial design: allocation of efficacy and process evaluation measures.

Abbreviations. GP: General Practitioner; PTSD: Post-Traumatic Stress Disorder; OCD: Obsessive-Compulsive Disorder; EEG: Electroencephalogram; NT: No Treatment; iCBT-I: Therapist-Guided Digital Cognitive Behavioral Therapy for Insomnia

**Appendix 1 - Interview guide of i-Sleep participants**

| **Interview guide i-Sleep participants** | | |
| --- | --- | --- |
| **Introduction** |  | **Introduction of the researcher:** Good morning/afternoon, this is [name] from the ‘Better Nights, Better Days?’ study. We had scheduled this interview now to discuss your experiences with i-Sleep. Does this still work for you?   **Purpose of the conversation:** First, thank you very much for participating in this evaluation. During your participation in the ‘Better Nights, Better Days?’ study, you went through the digital sleep module called i-Sleep. The purpose of this conversation is to evaluate your experiences with i-Sleep. We will use your feedback to optimize the i-Sleep module, aiming to eventually implement it in healthcare. There are no right or wrong answers—honesty is what helps us most, so please feel free to share openly.   **Permission to record the conversation:** With your permission, this conversation will be recorded and fully anonymized afterward. The recording will be transcribed verbatim, and the audio will then be deleted. Everything you share during this interview will not be traceable to you. Do you consent to the recording of this conversation? **If yes:** - Start the recording in the recording software. - Start ‘dictation’ in the Word file. - Just to confirm on the recording: do you, <nsr_xxxxx>, consent to the recording of this conversation? |
| **Questions** | | |
| **General questions about the study** |  | - Why did you sign up? What did you hope to achieve by participating in the study? (prompts: check questionnaire answers like reducing sleep problems, reducing anxiety symptoms, etc.) - How do you reflect on the information you received before the study started? - What expectations did you have about using i-Sleep?   - About the content of the module?   - About the outcomes of the module? |
| **General questions about the i-Sleep module** |  | After receiving all the information about participating in i-Sleep, you were able to start the program.   - Can you share your experiences with the i-Sleep module?  Why did you complete or not complete the module? |
| **Specific questions about the i-Sleep module (focusing on intervention elements)** |  | The following questions will focus on specific parts of the module, so I will give a brief overview of its structure. As you may recall, the module consisted of five sessions, each covering new topics. The first session addressed sleep hygiene, such as optimizing the bedroom environment, winding down your day, incorporating daytime rest moments, and lifestyle adjustments (e.g., increasing exercise, reducing caffeine and alcohol).  The second session introduced sleep restriction, where you reduced time in bed to increase sleep pressure. The third session taught techniques to deal with racing thoughts, like the “worry time,” thought-blocking exercises (repeating the word “the”), and relaxation exercises. The fourth session focused on reframing negative, unhelpful thoughts about sleep. The last session reflected on which parts of the module were effective for you and how to continue using them to improve your sleep.   - Which parts of the program did you use most? - Go through all the underlined components. - Which parts of the i-Sleep module did you find valuable? Can you name and explain an example? - What did you think of the use of example clients (Claudia, Frank, and Yasmin)? - Which parts of i-Sleep, in your opinion, had no added value? – - Was there anything you missed in the i-Sleep module that you would have liked to see included? – - What improvements would you suggest for the i-Sleep module? |
| **Digital method** |  | - What did you think of the online method used (the online sessions combined with digital feedback) during the i-Sleep module? (ask follow-up questions!) - Did you find the online sessions easy to use? Can you elaborate? - What went well or not so well? (logging in, completing sessions, asking for feedback, etc.) - What did you think of keeping the sleep log in the Sleep Log App? |
| **Therapeutic relationship and feedback** |  | - How was your experience with contact with your therapist? Why? - Was there anything you missed in your contact with the therapist? If yes, what was it? - What did you think of the content of the online feedback during the i-Sleep module? - Did you feel the online feedback sessions helped you reduce your symptoms?If yes, ask for specific sessions: “In session 4, adjusting negative thoughts was covered. Did the online conversations help with this? Or did you need more?”) - To what extent was the weekly feedback from the therapist necessary to understand the online sessions? Or did the online modules provide clarification for the weekly feedback? If yes, what was [un]clear? |
| **Experienced Effectiveness** |  | Now, let’s talk about the impact that following i-Sleep has had on you.   - Did the i-Sleep module meet your expectations? Why or why not? - Do you feel that i-Sleep helped you sleep better?   - If yes, how do you notice that? Can you give examples? (e.g., falling asleep faster, waking up less often during the night, sleeping through longer, feeling more rested during the day, etc.)   i-Sleep is primarily a sleep module, but we are also curious about its effects in other areas.   - Have you noticed effects of the module on... - ... your emotions, such as anxiety, depression, or other feelings? - ... your general daily mood? - ... your lifestyle? (physical activity, smoking, alcohol use, nutrition, etc.) - ... other areas? |
| **Continuation** |  | Now, let’s talk about whether you are still using i-Sleep or would like to use it in the future.   - Are you still using the information and techniques you learned from i-Sleep for your sleep?   - If yes, which ones and why? If not, why not? - Would you use i-Sleep again for similar complaints? - Would you recommend the i-Sleep module to someone else? |
| **Rounding off** | | |
| **Additional comments** |  | Those were all my questions for you. Is there anything else you’d like to share about the module that we haven’t discussed yet? Or do you have any additional comments or questions? |
| **Closing** |  | As I mentioned at the start, we will use your feedback to make improvements to the i-Sleep module, aiming for its eventual implementation in practice to alleviate insomnia. Your feedback will be pseudomized.  I’d like to thank you once again for participating in the ‘Better Nights, Better Days?’ study and for sharing your experiences with the i-Sleep module during this evaluation interview. Your insights are incredibly valuable to our research. Wishing you a great day! |

**Appendix 2 – Focus group interview guide for therapists**

| **Focus Group Interview Guide** | | |
| --- | --- | --- |
| **Introduction** |  | Good morning/afternoon, and thank you for agreeing to participate in this evaluation interview. My name is [name], and I am one of the researchers involved in the ‘Better Nights, Better Days?’ project. The goal of this interview is to evaluate your experiences with the digital module i-Sleep from your perspective as a therapist. Your feedback will help us optimize the i-Sleep module and the role of the therapist in it, so that the module may hopefully be implemented in the future. There are no right or wrong answers; the only thing that isn’t helpful is not being honest, as we rely on your genuine feedback. With your consent, this interview will be recorded, after which it will be completely anonymized. The recording will be transcribed literally, and the original recording will be deleted. Everything you share in this interview will be de-identified and not traceable back to you.   - Do you consent to the recording of this interview? [If yes, start recording] - Just to confirm on the recording: do you consent to the recording of this interview? |
| **Questions** | | |
| **Experience and expectations before guiding i-Sleep** |  | - Could you please introduce yourself? Who are you, and what is your role? - How did you hear about the BNBD study? - What led you to decide to work as a therapist in this study? - How much experience did you have with delivering i-Sleep or other sleep treatments before starting as a therapist in the ‘Better Nights, Better Days?’ study? - Had you previously worked with guiding people via an online module? If yes, how much? - What expectations did you have about guiding participants through i-Sleep? - About the content of the module? - About the therapeutic relationship with the patient? - About the practical aspects (online, scheduling, etc.)? - About its effectiveness? |
| **i-Sleep training** |  | Before you began treating participants using i-Sleep, you received training about it. This training consisted of a PowerPoint presentation covering the content of the i-Sleep treatment, background information on sleep and insomnia, and your role as a therapist. You also received tips on how to provide feedback to participants, and examples of what participants might have filled in during i-Sleep sessions and what suitable feedback might look like.   - What did you think of the training? - Which part of the training did you find most useful? Why? - Which part of the training did you find least useful? Why? - Did the training give you a clear understanding of how the online feedback should look? - Did you miss any information in the training? - Were there any parts of the training that you found unnecessary? |
| **Experiences with i-Sleep** |  | Next, you began treating participants.   - Could you describe how that went? (modules, providing feedback, participant drop-out, follow-up care, etc.) - Did you always follow the protocol? Why/why not? As you may recall, the i-Sleep module consists of 5 sessions. The first session covers psychoeducation and sleep hygiene. The second session focuses on stimulus control and sleep restriction. In the third session, relaxation exercises and reducing worrying are covered. In session 4, cognitive therapy is provided by adjusting unhelpful thoughts about sleep. The last session summarizes all prior sessions and includes relapse prevention. - What did you think of the module’s content?   - Did you feel that the information presented was useful?   - Are there any parts missing from the i-Sleep module?   - Would you change the structure/focus/order of the i-Sleep sessions? - How did you find the guidance you received while treating participants?   - What did you think of the supervision during the treatment of the first participants?   - What did you think of the weekly intervision (case-discussion) meetings? (frequency, content, usefulness?)   - To what extent did you feel it was possible to ask questions in between? - Based on your experience as a therapist, what is your impression of the effectiveness of the i-Sleep module in reducing sleep problems?   - Which parts do you feel most contribute to reducing sleep problems?   - Which parts do you think contribute less? - How did you experience the practical aspects of providing i-Sleep treatment?   - To what extent did you find it feasible to complete the 5 sessions of i-Sleep within 8 weeks?   For the participant?   - - - For you?   - What did you think of the user-friendliness of the online method used?     - For the participant?     - For you?   - What did you think of the frequency of feedback moments?   - What did you think of the format of the feedback moments? (written, via a single message)   - In terms of time investment, how would you rate the efficiency of i-Sleep compared to a face-to-face CBT-I treatment?   - Would you change anything in how the online guidance is provided during i-Sleep? - How did you experience building a therapeutic relationship during the i-Sleep treatment?   - Did you feel it was possible to build an adequate therapeutic relationship?   - Did the frequency of feedback play a role in building this relationship?   - Did the format of the feedback play a role in building this relationship? |
| **Continuation and implementation** |  | - If the therapist has stopped providing i-Sleep treatment: You stopped treating participants with i-Sleep in [month/year]. What was the reason for this? - How do you view the implementation of i-Sleep in practice?   - To what extent do you think it will be feasible to send reminders every week to participants who haven’t completed the session?   - Do you see yourself continuing as a therapist in this process?   - Would you recommend guiding i-Sleep to other therapists? |
| **Rounding off** | | |
| **Additional comments** |  | Is there anything else you would like to share about your experiences with the i-Sleep treatment that we haven’t discussed yet? |
| **Closing** |  | As I mentioned earlier, we will use your feedback to make improvements to the module, aiming for its eventual implementation in practice to alleviate insomnia. I want to thank you again for your contribution as a therapist and for sharing your experiences with i-Sleep in this conversation. Your insights are incredibly valuable for the research. Wishing you a great day! |

**Appendix 3 – Codebook**

| **RE-AIM dimension** |  | **Theme** |  | **Code** |  | **Explanation** |
| --- | --- | --- | --- | --- | --- | --- |
| **Reach** | | | | | | |
| Reasons for joining, continuing with, or opting out of the intervention among participants |  | Reasons for joining and expectations |  | Struggling for a long time |  | The participant has been experiencing sleep problems for a long time. |
|  |  |  |  | Best tip for better sleep |  | The participant has tried many things without success to improve their sleep and hopes to find the 'golden answer' in the i-Sleep module that will help them sleep better. |
|  |  |  |  | Scientific contribution |  | The participant wants to contribute to science through their participation. |
|  |  |  |  | No expectations |  | The participant had no expectations regarding the content of the module before starting i-Sleep. |
|  |  | Reasons to quit or continue with intervention |  | Participant quits |  | The participant did not complete the i-Sleep module and provides the reason(s) for this. |
|  |  |  |  | Adhering to i-Sleep |  | The participant completed the i-Sleep module and provides the reason(s) for this. |
| **Effectiveness** | | | | | | |
| Perceived effectiveness of the intervention on sleep quality, sleep environment and quality of life |  | Sleep hygiene |  | Good starting point |  | The session on sleep hygiene serves as a good introductory session because its content is not complex. |
|  |  |  |  | Added value |  | To what extent the session was beneficial for the participant |
|  |  |  |  | Negative effect |  | Sleep hygiene was either unsuccessful or had a negative impact on the participant. |
|  |  | Sleep restriction |  | Added value |  | To what extent the session was beneficial for the participant. |
|  |  |  |  | Negative effect |  | Sleep restriction was either unsuccessful or had a negative impact on the participant. |
|  |  | Managing worrying thoughts |  | Added value |  | To what extent the session was beneficial for the participant. |
|  |  |  |  | Negative effect |  | Applying the strategy for dealing with worrying thoughts ("the" strategy) was unsuccessful or had a negative impact on the participant. |
|  |  |  |  | Awareness |  | The worry exercises increased awareness |
|  |  | Relaxation exercises |  | Added value |  | To what extent the session was beneficial for the participant. |
|  |  |  |  | Negative effect |  | The relaxation exercises were unsuccessful or had a negative impact on the participant. |
|  |  | Changing negative thoughts about sleep |  | Added value |  | To what extent the session was beneficial for the participant. |
|  |  |  |  | Negative effect |  | Changing negative thoughts about sleep was unsuccessful or had a negative impact on the participant. |
|  |  | i-Sleep (in general) |  | Added value |  | To what extent the session was beneficial for the participant. |
|  |  |  |  | Content / format intervention |  | All feedback regarding the content and format of the intervention. |
|  |  |  |  | Duration |  | All comments about whether five weeks was an appropriate duration for completing the i-Sleep program. |
|  |  | Perceived effectiveness |  | Effect on sleep |  | All possible effects the i-Sleep module had on sleep, including both positive and negative outcomes. |
|  |  |  |  | Effect on emotions |  | All possible effects the i-Sleep module had on emotions, such as anxiety, sadness, or other emotional states. This includes both positive and negative outcomes. |
|  |  |  |  | Effect on lifestyle |  | All possible effects the i-Sleep module had on lifestyle, such as increased exercise, avoiding naps, or no effect. This includes both positive and negative outcomes. |
|  |  |  |  | Effect on awareness |  | All possible effects the i-Sleep module had awareness. This includes both positive and negative outcomes. |
| Determining dose response  analyses between  degree of adherence and  effectiveness |  | Sleep restriction |  | Application |  | The extent to which sleep restriction was applied |
|  |  |  |  | Suitability of target group |  | Sleep restriction is especially (not) suitable for certain groups, e.g. young people with irregular lifestyles. |
|  |  | Changing negative beliefs about sleep |  | Application |  | The extent to which learned strategies to change negative thoughts about sleep were applied |
|  |  | Sleep hygiene |  | Application |  | The extent to which learned strategies regarding sleep hygiene were applied |
|  |  | Managing worry (‘the’ strategy) |  | Application |  | The extent to which learned strategies to handle worrying thoughts were applied |
|  |  | Relaxation exercises. |  | Application |  | The extent to which learned relaxation exercises were applied |
|  |  | Sleep diary |  | Support |  | Feedback on whether the sleep diary and reminders were helpful as a motivating tool |
|  |  |  |  | Burden |  | Feedback on whether the sleep diary was burdensome or felt like too much effort for the participant. |
| Satisfaction, views, and experiences with the intervention among participants |  | Changing negative thoughts about sleep |  | Experience |  | How participants experienced the process of changing negative thoughts about sleep. |
|  |  | Example patients (case vignettes) |  | Added value |  | The participant found the use of example patients (case vignettes) to be a positive addition. |
|  |  |  |  | Negative / no added value |  | The participant found the use of example characters to be negative or felt it added no value |
|  |  | Sleep restriction |  | Experience |  | The participant's experience with the sleep restriction method. |
|  |  |  |  | New (unknown) element |  | The sleep restriction method was unfamiliar to the participant prior to the intervention. |
|  |  | Digital |  | Communication |  | The participant's experience with digital communication with the therapist. |
|  |  |  |  | Technical difficulties |  | Technical problems the participant encountered while completing the i-Sleep sessions. |
|  |  |  |  | Flexibility |  | The digital approach allows participants to choose the days on which they complete sessions, adding flexibility. |
|  |  |  |  | Added value of digital guidance |  | Any feedback regarding the contribution of online guidance to the intervention. |
|  |  | i-Sleep general |  | Starting Instructions |  | Feedback on the thoroughness and clarity of the instructions provided in the i-Sleep module. |
|  |  |  |  | depth |  | Participant feedback on the depth of the overall i-Sleep module, whether it was too detailed, too superficial, or just right. |
| **Adoption** |  |  |  |  |  |  |
| Reasons among supervising therapists/ experts for joining, continuing with, or opting out of using the intervention |  | Reason for opting out |  | Therapist stopped |  | The therapist provides the reason for deciding to stop participating in the intervention. |
|  |  | Reasons for participation |  | Scientific contribution |  | The therapist wishes to contribute to scientific research. |
|  |  |  |  | New adventure |  | The therapist became involved out of curiosity about a new element, target group, or experience. |
|  |  |  |  | Involved in effectiveness study |  | The therapist became involved because they assisted with the RCT as researcher. |
| **Implementation** |  |  |  |  |  |  |
| Treatment adherence among participants, e.g., completed sessions and assignments (fidelity) |  | Following treatment by participants |  | Completing i-Sleep |  | Whether the participant completed the i-Sleep module and the reasons for doing so. |
| Degree in which the intervention protocol has been followed by therapists (fidelity and dosage) |  | Manual/protocol |  | Therapists following the research protocol |  | All information regarding how closely the therapists adhered to the research protocol. |
|  |  | Feedback templates |  | use of Feedback templates |  | All information regarding how therapists used the feedback templates. |
| Use (dosage and fidelity) of provided training/support regarding the intervention among therapists |  | Training for i-Sleep therapists |  | Experience |  | All information about how therapists experienced the training prior to starting i-Sleep. |
|  |  |  |  | Applying in the ‘Real World’ |  | All information about how therapists experienced giving feedback after receiving i-Sleep training. |
|  |  | Support among therapists/supervisors |  | Case discussion meetings/Supervision |  | All information shared by therapists regarding case discussion meetings and supervision. |
|  |  |  |  | Support outside case-discussion meetings/supervision |  | All information shared by therapists regarding the support they received outside of peer meetings and supervision. |
| Satisfaction, views and experiences with the intervention and provided training/support among therapists |  | ICT Problems |  | Sleep diary Issues |  | Problems with the sleep diary identified by therapists, such as lack of an overview of sleep data or the need to manually calculate hours. |
|  |  |  |  | Other ICT Issues |  | Any other technical problems therapists encountered. |
|  |  | i-Sleep general |  | Advantages |  | All benefits of the i-Sleep module from the perspective of the therapist |
|  |  |  |  | disadvantages |  | All drawbacks of the i-Sleep module from the perspective of the therapist |
| **Maintenance** |  |  |  |  |  |  |
| Long-term helpfulness and continued usage of intervention elements among participants |  | Experienced effectiveness |  | Effect on Sleep |  | All possible long-term effects the i-Sleep module had on sleep, such as easier falling asleep, no effect, improved sleep/wake rhythm, or uncertainty about the effect. |
|  |  |  |  | Effect on emotions |  | All possible long-term effects the i-Sleep module had on emotions, such as anxiety, sadness, or other emotions, or no effect. |
|  |  |  |  | Effect on lifestyle |  | All possible long-term effects the i-Sleep module had on lifestyle, such as more exercise, no naps, or no effect. |
|  |  |  |  | Effect on awareness |  | Completing the i-Sleep module led to long-term greater awareness. |
|  |  |  |  |  |  |  |
| Intention for long-term use of the intervention among therapists |  | Disadvantages of (Online) Module |  | Not Tailored |  | All disadvantages regarding the lack of tailored, individualized care, where the severity of the complaints might require more or less care for a person. |
|  |  |  |  | Not Face-to-Face |  | All disadvantages related to the absence of face-to-face contact between the therapist and participant. |
|  |  |  |  | Motivation / accountability |  | All disadvantages related to the difficulty of motivating participants via the online module. |
|  |  |  |  | Communication between therapist and participant: |  | All disadvantages related to the quantity and quality of communication between the therapist and participant. |
|  |  |  |  | Time |  | All disadvantages noted by therapists regarding the time or duration of the module and sessions. |
|  |  | Advantages of the (Online) Module |  | therapist satisfaction |  | All information regarding how much satisfaction the therapist derives from guiding a participant. |
| Determining future activities and intention to use the intervention |  | Future/Preventive |  | Continued application |  | Whether the participant continues to apply the strategies learned in the i-Sleep module to improve sleep. |
|  |  |  |  | Relapse into old behavior |  | Whether the participant finds it difficult to maintain the learned strategies and sometimes relapses. |
|  |  |  |  | Reverting to effective elements |  | Whether the participant will reapply the elements of i-Sleep that were effective when sleep issues return. |
|  |  |  |  | Recommending to others |  | Whether the participant plans to recommend the i-Sleep module to others in the future. |
| **Overall** | | | | | | |
| Identifying recommendations for improvement of the intervention and implementation support beyond the research setting |  | Tips/Suggestions |  | More Information from therapist |  | Any tips or suggestions regarding information provided by therapists. |
|  |  |  |  | Module functionality |  | Any tips or suggestions regarding the functions within the online module. |
|  |  |  |  | Communication between therapist and participant |  | Any tips or suggestions related to the communication between the therapist and participant. |

**Appendix 4 – COREQ checklist**


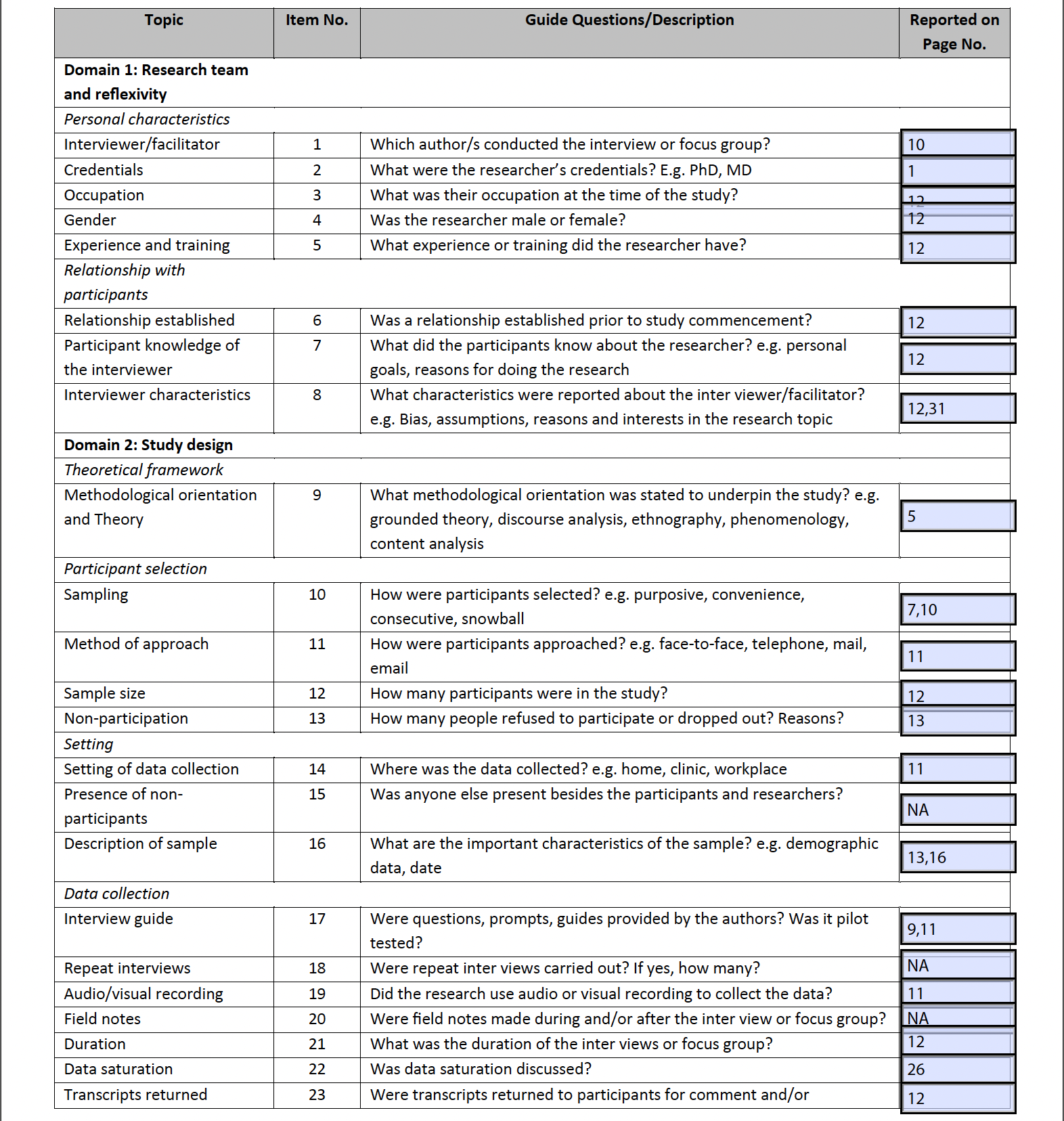

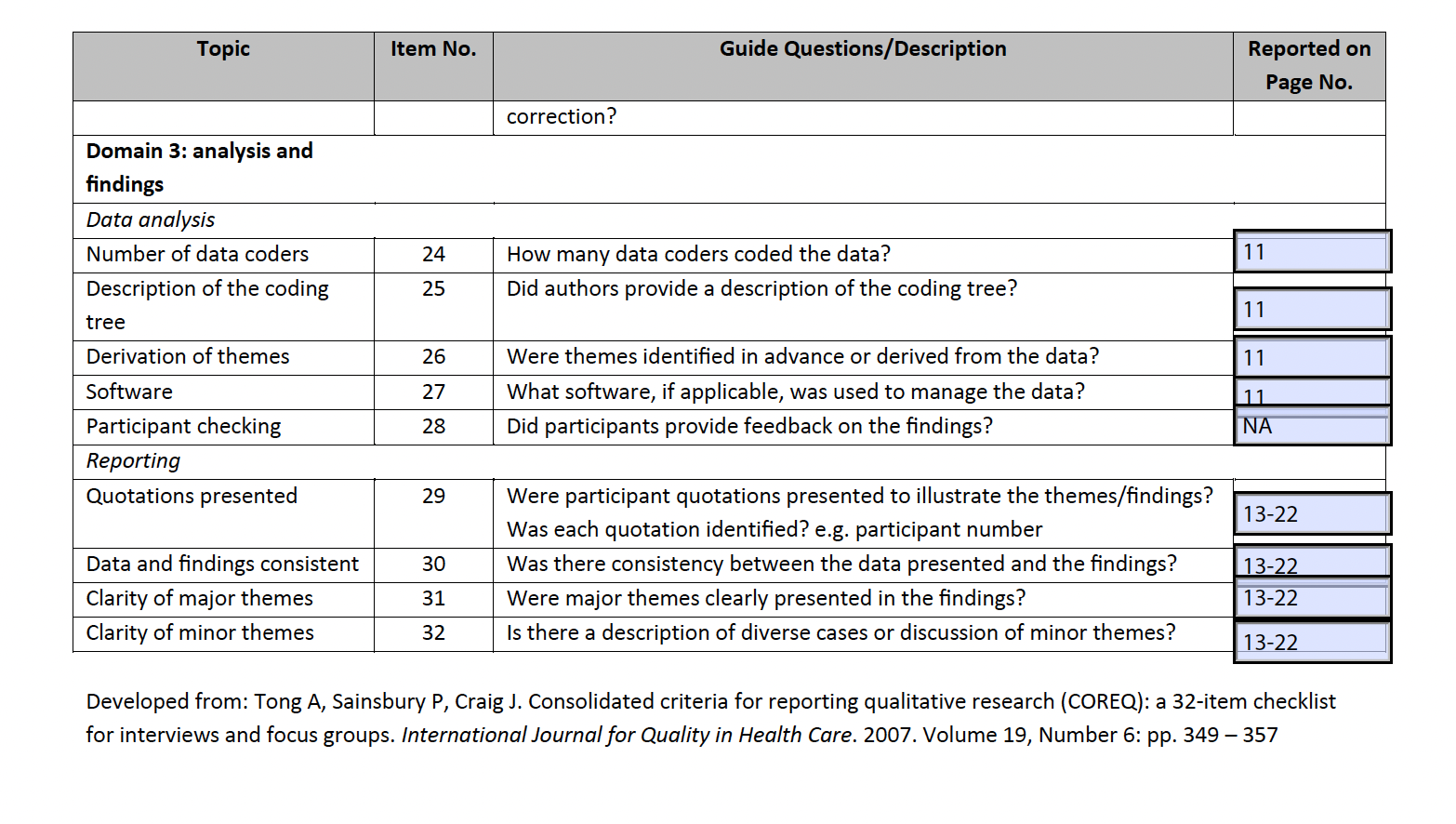


**Appendix 5 - Overview of all administered process evaluation questionnaires, including specific items and scales**

| **Process evaluation questionnaire - therapists** | | |
| --- | --- | --- |
| **Item** |  | **Response option** |
| What is your biological sex assigned at birth? |  | ( ) Man, ( ) Woman |
| What is your birth year |  | Open text field |
| What is your country of birth? |  | ( ) Netherlands, ( ) Suriname, ( ) Morocco, ( ) Turkey, ( ) Antilles, ( ) Other: ________ |
| What is your mother’s country of birth? |  | ( ) Netherlands, ( ) Suriname, ( ) Morocco, ( ) Turkey, ( ) Antilles, ( ) Other: ________ |
| What is your father’s country of birth? |  | ( ) Netherlands, ( ) Suriname, ( ) Morocco, ( ) Turkey, ( ) Antilles, ( ) Other: ________ |
| What is your highest completed education? |  | ( ) No education, ( ) Primary education, ( ) VMBO, ( ) HAVO, ( ) VWO, ( ) MBO, ( ) HBO, ( ) WO, ( ) Other: ________ |
| What is your current clinical function? |  | [ ] Base psychologist, [ ] GZ psychologist, [ ] Clinical (neuro) psychologist, [ ] Psychotherapist, [ ] Psychiatric nurse, [ ] Psychiatrist, [ ] Other: ________ |
| How many years of clinical experience (in mental health care) do you have? |  | Open text field |
| Do you have previous experience with i-Sleep module? |  | ( ) No, ( ) Yes |
| If yes, what was your experience with i-Sleep? |  | Scale: 1 (very dissatisfied) to 10 (very satisfied |
| Do you have experience with other sleep treatment programs |  | ( ) No, ( ) Yes |
| What are your reasons for participating in guiding i-Sleep participants in this research setting? (multiple options) |  | [ ] Expand knowledge about sleep and insomnia, [ ] Help patients with sleep problems, [ ] Contribute to scientific knowledge, [ ] Requested by supervisor, [ ] Requirement for training, [ ] Recommended by colleague, [ ] Other: ________ |
| How did you hear about the research? (multiple options) |  | [ ] Supervisor, [ ] Colleague, [ ] Researcher, [ ] Institution’s website/intranet, [ ] Other: ________ |
| *How do you view the use of the online i-Sleep module for treating sleep problems in people with anxiety disorders and borderline personality disorder? Indicate to what extent you agree with the following statements.* | | |
| *"I find it important to address sleep problems with my patients."* |  | Scale: 1 (Strongly disagree) to 5 (Strongly agree) |
| *“I feel responsible for addressing sleep issues with my patients."* |  |  |
| *"My colleagues believe it is important to address sleep problems with patients."* |  |  |
| *"I have sufficient knowledge to address sleep problems with my patients."* |  |  |
| *"I have sufficient skills to address sleep problems with my patients."* |  |  |
| *"I think addressing sleep problems can positively affect a patient's daily functioning."* |  |  |
| *"I am motivated to address sleep problems with my patients."* |  |  |
| *“Addressing sleep problems with patients aligns with my team's policy."* |  |  |
| *“I have enough time to address sleep problems with my patients."* |  |  |
| To what extent didyou feel prepared from training for delivering i-Sleep? |  | Scale: 1 (completely unprepared) to 10 (very well prepared) |
| Which aspects of the training you found useful? |  | Open text field |
| Which aspects of the training you found less useful? |  | Open text field |

| **Process evaluation questionnaire – participants (T1)** | | |
| --- | --- | --- |
| **Item** |  | **Response option** |
| Have you completed all sessions of the i-Sleep online module? |  | ( ) No, I stopped the i-Sleep online module early  ( ) No, I am still working on completing the online sessions  ( ) Yes |
| How satisfied were you with the i-Sleep online module? Rate your satisfaction on a scale of 1 (very dissatisfied) to 10 (very satisfied). |  | 1 (very dissatisfied) to 10 (very satisfied) |
| Was the personal guidance you received during the i-Sleep online module sufficient? |  | ( ) No  ( ) Yes |
| What did you feel was missing from the personal guidance during the i-Sleep online module? |  | Open text field |
| Were the various topics, advice, and homework assignments clearly explained in the i-Sleep online module? |  | ( ) No  ( ) Yes |
| Which topics, advice, and/or homework assignments were insufficiently explained? |  | Open text field |
| *How often did you apply the following advice and homework assignments during the i-Sleep online module?* | | |
| Adjusting lifestyle (alcohol, exercise, relaxation during the day, etc.) |  | Scale: 1 (never performed) to 10 (performed daily) |
| Adjusting your bedroom environment (light, sound, comfort, etc.) |  |  |
| Gradually winding down your day (fixed evening ritual) |  |  |
| Applying sleep restriction (limiting time spent in bed) |  |  |
| Maintaining a fixed day-night rhythm (consistent bedtimes) |  |  |
| Implementing a worry time |  |  |
| Completing additional worry exercises (blocking thoughts, etc.) |  |  |
| Performing relaxation exercises (muscle exercises, etc.) |  |  |
| Adjusting incorrect, unhelpful thoughts about sleep and insomnia (creating alternatives) |  |  |
| *How feasible was it to apply the following advice and complete the homework assignments daily? Rate this on a scale of 1 (not feasible) to 10 (very feasible).* | | |
| Adjusting lifestyle (alcohol, exercise, relaxation during the day, etc.) |  | Scale: NA (not applicable) to 10 (highly feasible) |
| Adjusting your bedroom environment (light, sound, comfort, etc.) |  |  |
| Gradually winding down your day (fixed evening ritual) |  |  |
| Applying sleep restriction (limiting time spent in bed) |  |  |
| Maintaining a fixed day-night rhythm (consistent bedtimes) |  |  |
| Implementing a worry time |  |  |
| Completing additional worry exercises (blocking thoughts, etc.) |  |  |
| Performing relaxation exercises (muscle exercises, etc.) |  |  |
| Adjusting incorrect, unhelpful thoughts about sleep and insomnia (creating alternatives) |  |  |
| *How effective were the following advice and techniques from the i-Sleep online module in reducing your sleep problems?* | | |
| Adjusting lifestyle (alcohol, exercise, relaxation during the day, etc.) |  | Scale: 1 (not at all effective) to 10 (highly effective) |
| Adjusting your bedroom environment (light, sound, comfort, etc.) |  |  |
| Gradually winding down your day (fixed evening ritual) |  |  |
| Applying sleep restriction (limiting time spent in bed) |  |  |
| Maintaining a fixed day-night rhythm (consistent bedtimes) |  |  |
| Implementing a worry time |  |  |
| Completing additional worry exercises (blocking thoughts, etc.) |  |  |
| Performing relaxation exercises (muscle exercises, etc.) |  |  |
| Adjusting incorrect, unhelpful thoughts about sleep and insomnia (creating alternatives) |  |  |
| Creating future plan (evaluating and continuing learned techniques) |  |  |
| *Give your opinion on your therapist who guided you during the i-Sleep online module, using a scale of 1 (strongly disagree) to 10 (strongly agree):* | | |
| My therapist gave me good advice. |  | Scale: 1 = completely disagree to 10 (completely agree) |
| My therapist took my personal circumstances into account. |  |  |
| My therapist was easily reachable for questions. |  |  |
| My therapist gave me choices and options. |  |  |
| My therapist indicated what I should do to make progress. |  |  |
| My therapist motivated me to complete the online treatment. |  |  |
| My therapist gave me confidence in addressing my sleep problems. |  |  |
| My therapist took the time for me. |  |  |
| Do you have suggestions for improving the i-Sleep online module? |  | ( ) No  ( ) Yes |
| What are your suggestions for improving the i-Sleep online module? |  | Open text field |
| Do you have suggestions for improving the guidance in the i-Sleep online module? |  | ( ) No  ( ) Yes |
| What are your suggestions for improving the guidance of the i-Sleep online module? |  | Open text field |
| Would you recommend the i-Sleep online sleep treatment, including online guidance from a therapist to fellow patients who are also struggling with sleep problems? |  | ( ) No  ( ) Yes |
| Do you have any additional comments or suggestions to improve i-Sleep? (optional) |  | Open text field |

| **Process evaluation questionnaire – participants (T2)** | | |
| --- | --- | --- |
| **Item** |  | **Response option** |
| *To what extent have you carried out the following activities since completing/finishing the online sleep treatment? Rate on a scale from 1 (never done again) to 10 (done daily):* | | |
| Reflecting on your lifestyle and making necessary changes (alcohol use, exercise, daytime relaxation, etc.) |  | Scale: 1 (never performed) to 10 (performed daily) |
| Reflecting on your bedroom environment and making necessary changes (light, noise, comfort, etc.) |  |  |
| Gradually winding down the day with a fixed evening routine |  |  |
| Limiting the time spent in bed to increase sleep pressure |  |  |
| Maintaining regular bedtimes and wake-up times |  |  |
| Doing worry exercises to reduce worrying thoughts (worry time, etc.) |  |  |
| Performing relaxation exercises to ensure sufficient relaxation during the day |  |  |
| Reflecting on incorrect thoughts about sleep and insomnia, and formulating necessary alternative thoughts |  |  |
| Referring to and following the future plan you created |  |  |
| Have you done any other activities since completing the online sleep treatment to reduce your sleep problems? |  | ( ) No  ( ) Yes |
| What other activities have you done to reduce your sleep problems since completing the online sleep treatment? |  | Open text field |
| Do you have any further comments or suggestions to improve i-Sleep? (optional) |  | Open text field |
